# Supplementary material for: Carbon opportunity cost increases carbon footprint advantage of grain-finished beef
Source: PLoS One. 2023 Dec 13;18(12):e0295035. doi: 10.1371/journal.pone.0295035 (PMC10718409; doi:10.1371/journal.pone.0295035)
Supplement: S1 File — (DOCX) [file pone.0295035.s001.docx]

**Supporting Information for**

Carbon Opportunity Cost Increases Carbon Footprint Advantage of Grain-Fed Beef

**This PDF file includes:**

Supplementary Methods

SI References

S1 Fig

S1 to S8 Tables

## Supplementary Methods

### Selection of Pasture-Finished/Grain-Finished Pairs

We categorized beef-producing operations included in the Poore & Nemecek (2018)^1^ [“Full Excel model” dataset](https://ora.ox.ac.uk/objects/uuid:a63fb28c-98f8-4313-add6-e9eca99320a5) as pasture-finished or grain-finished. We categorize operations as pasture-finished when they are described as grass-fed, grass-finished, pasture-based or extensive in the dataset or in the original study. When no distinction was made, we categorized operations as pasture-finished if the original study did not state cattle were fed grain and if permanent pasture accounted for 94% or more of land use (Seed + Farm + Fallow) in the Poore & Nemecek (2018) dataset. Operations were otherwise classified grain-finished.

When there were multiple pasture-finished or grain-finished operations in the same region or from the same study that could be compared, to be conservative we chose the two with the most similar land use intensities.

### Potential Carbon Sequestration

To calculate potential carbon sequestration we used the following data sources:

- A csv of latitude and longitude for each operation drawn from Poore & Nemecek (2018) except where noted below.
- Raster data on annual net primary productivity (NPP) of potential native vegetation from Searchinger et al (2018)^2^ and available as “lpjml_anpp_avg_2001-2010.asc” from <https://doi.pangaea.de/10.1594/PANGAEA.893761?format=html#download>
- Raster data of croplands and rangeland classified into Anthropogenic Biomes (Anthromes v2) from Hyde 3.2.1.^3^

We conducted the following analysis in QGIS 3.10.5:

- Separately masked the NPP raster layer with the croplands and rangeland layers and set values of zero to NA in order to eliminate values for non-agricultural land from further calculations.
- Created buffers of size 0.25, 0.5, 1.0., 2.0 and 4.0, with other buffer options set to default values.
- Calculated the mean NPP value on croplands and rangeland for each buffer using Zonal Statistics.

Several operations lacked latitudes and longitudes. We included several of these in the comparison of pasture and grain-finished operations. To calculate potential carbon sequestration for these operations, we calculated the mean NPP value for the country or subnational region the operation was associated with. Regions used for each operation are listed below. For the Cerrado, we used a shapefile developed by the Brazilian Ministry of Environment and the Brazilian Institute of Geography and Statistics, available from the World Resources Institute [here](https://data.globalforestwatch.org/datasets/54ec099791644be4b273d9d8a853d452_4). For all other regions, we used country and state/province shapefiles from Natural Earth (1:10m v4.1.0).

**Regions Used to Calculate Potential Carbon Sequestration for Pasture- and Grain-Finished Operations Lacking Lat/Lon**

| **Study** | **Region** |
| --- | --- |
| Alig et al. (2012) | Switzerland |
| Capper (2012) | USA |
| Cardoso et al. (2016) | Cerrado, Brazil |
| Casey and Holden (2006a); Blonk et al. (2008) | Ireland |
| Mogensen et al. (2015) | Denmark; Sweden |
| Pashei Kamali et al. (2016) | Southern Brazil |
| Pelletier et al. (2010) | Iowa, USA |
| Picasso et al. (2014) | Uruguay |
| Stanley et al. (2018) | Michigan, USA |
| Wiedemann et al. (2015b) | New South Wales & Queensland, Australia |

### Standardizing studies

The Poore & Nemecek (2018) database only lists cropland land area for Pelletier et al. (2010)^4^. We allocate this to cropland and grazing by multiplying the land area for the two operations by the percentage of land occupied by cow-calf and finishing stages, as reported in Pelletier (2010).

We include one study in our analysis, Stanley et al. (2018)^5^, that was not included in Poore & Nemecek (2018). To standardize the data with that presented in Poore & Nemecek (2018), we impute several values using data from Pelletier et al. (2010) as this study was conducted in the most similar region. We:

- Calculate pasture land area in the cow-calf stage by dividing average weight at the end of the cow-calf stage, as reported in Stanley et al. (2018), by the average land use per calf in the cow-calf stage, as reported by Pelletier et al. (2010).
- Calculate total pasture area use by adding land area used in the cow-calf stage to land area used during the finishing stage, as reported in Stanley et al. (2018).
- Calculate total production emissions by dividing the average emissions per calf from the cow-calf stage reported in Pelletier et al. (2010) by the average weight at the end of the cow-calf stage, as reported in Stanley et al. (2018), and then adding the result to the carbon footprint for the finishing stage reported in Stanley et al. (2018).
- Convert the functional weight for values from hot-standard carcass weight to retail weight using a multiplier of 0.72, as used in Poore & Nemecek (2018).
- Categorize the climate as “Cool Temperature, Moist.”

### Robustness Checks

We vary four parameters to assess the robustness of the results.

Radius: We calculate mean NPP on cropland and rangeland within a 0.25, 0.5, 1.0, 2.0 and 4.0 degree radius of the latitude/longitude listed in Poore & Nemecek (2018) for each included operation.

Carbon Opportunity Cost (COC): In addition to calculating COC based on NPP values within a set radius, we calculate national COC using the average NPP values over all crop and rangeland across the country each operation is located in. We used country shapefiles from Natural Earth (1:10m v4.1.0). One limitation of this approach is that we do not estimate separate potential carbon sequestration or COC values for pasture, rye, alfalfa, corn, soy, and other specific land uses. This is because many of the beef LCAs used do not list specific crop types. Given that the COC attributed to grazing land use accounts for the majority of the total COC for beef production, we do not expect that calculating crop-specific values would substantially affect our results. In addition, our estimates are not weighted by the level of crop or livestock production across crop and rangeland.

We also calculate global COC using the average NPP values over all crop and rangeland globally, as categorized into Anthropogenic Biomes (Anthromes v2) from Hyde 3.2.1. These estimates share the same limitations as the national COC estimates.

Carbon Sequestration Rate: We estimate carbon sequestration potential for operations using two global values. In the primary results, we use a value of 0.28 MgC ha^-1^ yr^-1^ for grazing practices estimated in Conant et al. (2017)^6^. These include changes in grazing intensity, fertilization, and grasses. As a robustness check, we calculate results with a value of 0.47 MgC ha^-1^ yr^-1^. This is the value reported for all “improved” grassland management practices from Conant et al. (2017). These include changes in irrigation and stock rates, legume sowing, removal of grazing livestock, rotation grazing, season grazing, among other practices.

Although these global average values are inherently imprecise for assessing carbon sequestration from individual operations, we consider the values reasonable to use. They fall within the range of published estimates^7^. To be conservative in our carbon footprint for GF operations, we assume that no soil C sequestration occurs on cropland used for feed production, consistent with research that CO_2_ emissions from agricultural land are generally balanced by removals.^8^

Fourth, we ran the analysis with (yes) and without (no) the potential carbon sequestration, and thus the COC, set to 0 for operations in dry climates.

## SI References

1. Poore, J. & Nemecek, T. Reducing food’s environmental impacts through producers and consumers. *Science (80-. ).* **360**, 987–992 (2018).

2. Searchinger, T. D., Wirsenius, S., Beringer, T. & Dumas, P. Assessing the efficiency of changes in land use for mitigating climate change. *Nature* (2018). doi:10.1038/s41586-018-0757-z

3. Klein Goldewijk, K., Beusen, A., Doelman, J. & Stehfest, E. Anthropogenic land use estimates for the Holocene – HYDE 3.2. *Earth Syst. Sci. Data* **9**, 927–953 (2017).

4. Pelletier, N., Pirog, R. & Rasmussen, R. Comparative life cycle environmental impacts of three beef production strategies in the Upper Midwestern United States. *Agric. Syst.* **103**, 380–389 (2010).

5. Stanley, P. L., Rowntree, J. E., Beede, D. K., DeLonge, M. S. & Hamm, M. W. Impacts of soil carbon sequestration on life cycle greenhouse gas emissions in Midwestern USA beef finishing systems. *Agric. Syst.* **162**, 249–258 (2018).

6. Conant, R. T., Cerri, C. E. P. P., Osborne, B. B. & Paustian, K. Grassland management impacts on soil carbon stocks: A new synthesis: A. *Ecol. Appl.* **27**, 662–668 (2017).

7. Garnett, T. *et al.* *Grazed and confused? Ruminating on Cattle, Grazing Systems, Methane, Nitrous Oxide, the Soil Carbon Sequestration Question-and what it All Means for Greenhouse Gas Emissions.* (Food Climate Research Network, 2017).

8. Smith, P. *et al.* Agriculture, Forestry and Other Land Use (AFOLU). in *Climate Change 2014: Mitigation of Climate Change. Contribution of Working Group III to the Fifth Assessment Report of the Intergovernmental Panel on Climate Change* (eds. Edenhofer, O., R., Pichs-Madruga, Y. Sokona, E. Farahani, S. Kadner, K. Seyboth, A. Adler, I. Baum, S. Brunner, P. Eickemeier, B. Kriemann, J. & Savolainen, S. Schlömer, C. von Stechow, T. Z. and J. C. M.) (Cambridge University Press, 2014).

## Supplementary Figures

## S1 Fig: Mean potential carbon sequestration within 2 degrees of geocoded LCAs.


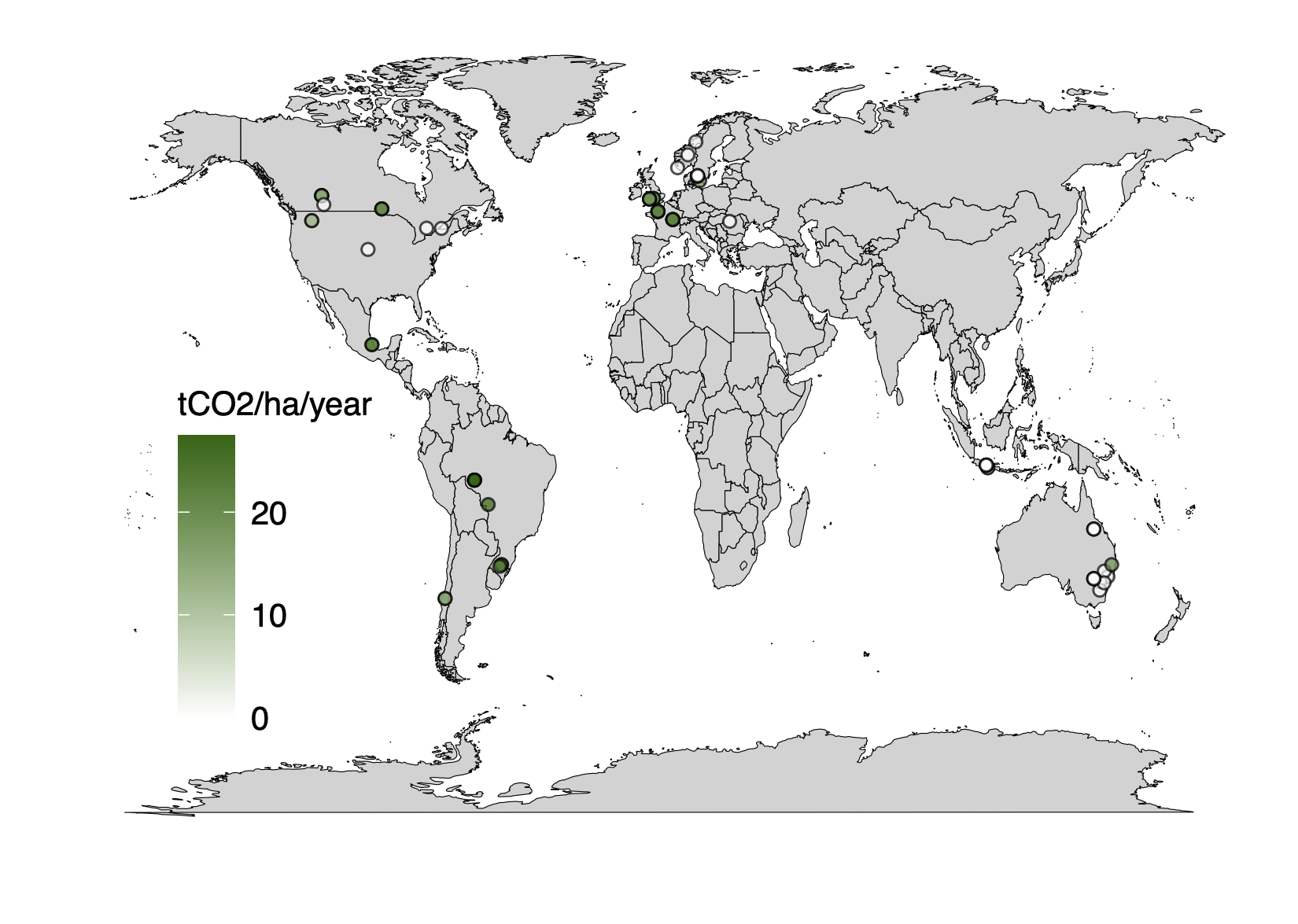


White circles indicate operations in dry climates, where potential carbon sequestration is set to 0. N = 72. Made with Natural Earth, a public domain map dataset.

## Supplementary Tables

S1 Table: Mean Carbon Opportunity Cost (COC), Production Emissions (PEM) and Soil Carbon Sequestration (SCS) of Operations included in Regression & Comparison

S2 Table: Mean Differences Between Carbon Opportunity Cost (COC), Production Emissions (PEM) and Soil Carbon Sequestration (SCS) Across All Operations

S3 Table: Pasture-Finished Operations Relative to Grain-Finished Operations

S4 Table: Difference between Pasture- and Grain-Finished Carbon Footprint (kgCO_2_e kg^-1^) under Robustness Checks

S5 Table: Regression Results for Pooled and Fixed Effects Specifications, Radius = 2.0

S6 Table: Beta Coefficient from Log-Log Regression of Carbon Footprint on Land Use Intensity with a Random Effect Specification Under Robustness Checks

S7 Table: Studies Included in Regression Analysis

S8 Table: Studies Included in Pasture-Finished (PF) / Grain-Finished (GF) Comparison

#### S1 Table: Mean Carbon Opportunity Cost (COC), Production Emissions (PEM) and Soil Carbon Sequestration (SCS) of Operations included in Regression & Comparison

| **Dry** | **SCS  (MgC ha^-1^ yr^-1^)** | **Radius (degrees)** | **COC (kgCO_2_e kg^-1^)** | **SCS  (kgCO_2_e kg^-1^)** | **PEM  (kgCO_2_e kg^-1^)** | **PEM + SCS + COC  (kgCO_2_e kg^-1^)** |
| --- | --- | --- | --- | --- | --- | --- |
| yes | 0.28 | 0.25 | 104.1 | 15.1 | 52.6 | 141.6 |
| yes | 0.28 | 0.5 | 111.2 | 15.1 | 52.6 | 148.8 |
| yes | 0.28 | 1 | 124.3 | 15.1 | 52.6 | 161.8 |
| yes | 0.28 | 2 | 139.8 | 15.1 | 52.6 | 177.4 |
| yes | 0.28 | 4 | 139.8 | 15.1 | 52.6 | 177.3 |
| yes | 0.47 | 0.25 | 104.1 | 25.4 | 52.6 | 131.3 |
| yes | 0.47 | 0.5 | 111.2 | 25.4 | 52.6 | 138.5 |
| yes | 0.47 | 1 | 124.3 | 25.4 | 52.6 | 151.6 |
| yes | 0.47 | 2 | 139.8 | 25.4 | 52.6 | 167.1 |
| yes | 0.47 | 4 | 139.8 | 25.4 | 52.6 | 167.1 |
| no | 0.28 | 0.25 | 136.2 | 15.1 | 52.6 | 173.7 |
| no | 0.28 | 0.5 | 144.5 | 15.1 | 52.6 | 182 |
| no | 0.28 | 1 | 158.9 | 15.1 | 52.6 | 196.5 |
| no | 0.28 | 2 | 172.5 | 15.1 | 52.6 | 210 |
| no | 0.28 | 4 | 168.2 | 15.1 | 52.6 | 205.7 |
| no | 0.28 | Global | 98.3 | 15.1 | 52.6 | 135.8 |
| no | 0.28 | National | 154.3 | 15.1 | 52.6 | 191.8 |
| no | 0.47 | 0.25 | 136.2 | 25.4 | 52.6 | 163.5 |
| no | 0.47 | 0.5 | 144.5 | 25.4 | 52.6 | 171.7 |
| no | 0.47 | 1 | 158.9 | 25.4 | 52.6 | 186.2 |
| no | 0.47 | 2 | 172.5 | 25.4 | 52.6 | 199.8 |
| no | 0.47 | 4 | 168.2 | 25.4 | 52.6 | 195.5 |
| no | 0.47 | Global | 98.3 | 25.4 | 52.6 | 125.6 |
| no | 0.47 | National | 154.3 | 25.4 | 52.6 | 181.6 |

#### S2 Table: Mean Differences Between Carbon Opportunity Cost (COC), Production Emissions (PEM) and Soil Carbon Sequestration (SCS) Across All Operations

| **Dry** | **SCS** | **Radius** | **COC / PEM** | **SCS / PEM** |
| --- | --- | --- | --- | --- |
| yes | 0.28 | 0.25 | 1.65 | 0.31 |
| yes | 0.28 | 0.5 | 1.74 | 0.31 |
| yes | 0.28 | 1 | 1.96 | 0.31 |
| yes | 0.28 | 2 | 2.30 | 0.31 |
| yes | 0.28 | 4 | 2.31 | 0.31 |
| yes | 0.47 | 0.25 | 1.65 | 0.53 |
| yes | 0.47 | 0.5 | 1.74 | 0.53 |
| yes | 0.47 | 1 | 1.96 | 0.53 |
| yes | 0.47 | 2 | 2.30 | 0.53 |
| yes | 0.47 | 4 | 2.31 | 0.53 |
| no | 0.28 | 0.25 | 2.53 | 0.31 |
| no | 0.28 | 0.5 | 2.66 | 0.31 |
| no | 0.28 | 1 | 2.92 | 0.31 |
| no | 0.28 | 2 | 3.20 | 0.31 |
| no | 0.28 | 4 | 3.10 | 0.31 |
| no | 0.28 | Global | 2.03 | 0.31 |
| no | 0.28 | National | 2.77 | 0.31 |
| no | 0.47 | 0.25 | 2.53 | 0.53 |
| no | 0.47 | 0.5 | 2.66 | 0.53 |
| no | 0.47 | 1 | 2.92 | 0.53 |
| no | 0.47 | 2 | 3.20 | 0.53 |
| no | 0.47 | 4 | 3.10 | 0.53 |
| no | 0.47 | Global | 2.03 | 0.53 |
| no | 0.47 | National | 2.77 | 0.53 |

#### S3 Table: Pasture-Finished Operations Relative to Grain-Finished Operations

| **Variable** | **Mean Difference** | **Mean Percent Difference** | **P-Value (Paired T-Test)** | **Median Difference** | **Median Percent Difference** | **P-Value (Wilcoxon Signed Rank)** |
| --- | --- | --- | --- | --- | --- | --- |
| PEM | 6.219 | 19.951 | 0.0002 | 5.933 | 15.357 | 0.0001 |
| PEM + SCS | 0.352 | 5.659 | 0.8364 | -2.329 | -5.345 | 0.5217 |
| PEM + COC | 43.088 | 43.239 | 0.0044 | 12.083 | 19.443 | 0.0014 |
| PEM + SCS + COC | 37.221 | 41.982 | 0.0083 | 10.674 | 14.889 | 0.0107 |
| Land-use intensity | 0.005 | 56.998 | 0.0007 | 0.004 | 26.727 | 0.0004 |

Positive (negative) values indicate higher (lower) mean and median values for pasture-finished operations than for grain-finished operations. Units for mean and median land-use intensity are ha/kg retail weight. Units for production emissions (PEM), soil carbon sequestration (SCS) and carbon opportunity cost (COC) are kg CO_2_e kg**^-1^** retail weight. *n* = 20 pairs.

#### S4 Table: Difference between Pasture- and Grain-Finished Carbon Footprint (kg CO_2_e kg^-1^) under Robustness Checks

|  |  |  | **Mean** | | | **Median** | | |
| --- | --- | --- | --- | --- | --- | --- | --- | --- |
| **Dry** | **SCS** | **Radius/COC Type** | **PEM** | **PEM + SCS** | **PEM + SCS + COC** | **PEM** | **PEM + SCS** | **PEM + SCS + COC** |
| yes | 0.28 | 0.25 | 6.2*** | 0.4 | 21.6* | 6.2*** | -1.5 | 16.1 |
| yes | 0.28 | 0.5 | 6.2*** | 0.4 | 21.6* | 6.2*** | -1.5 | 16 |
| yes | 0.28 | 1 | 6.2*** | 0.4 | 27.2** | 6.2*** | -1.5 | 18.1* |
| yes | 0.28 | 2 | 6.2*** | 0.4 | 37.2*** | 6.2*** | -1.5 | 26.6** |
| yes | 0.28 | 4 | 6.2*** | 0.4 | 35.7*** | 6.2*** | -1.5 | 25.6** |
| yes | 0.47 | 0.25 | 6.2*** | -3.6 | 17.7 | 6.2*** | -5.5* | 11.4 |
| yes | 0.47 | 0.5 | 6.2*** | -3.6 | 17.7 | 6.2*** | -5.5* | 11.5 |
| yes | 0.47 | 1 | 6.2*** | -3.6 | 23.2* | 6.2*** | -5.5* | 13.7 |
| yes | 0.47 | 2 | 6.2*** | -3.6 | 33.2** | 6.2*** | -5.5* | 24.9** |
| yes | 0.47 | 4 | 6.2*** | -3.6 | 31.7** | 6.2*** | -5.5* | 23.6** |
| no | 0.28 | 0.25 | 6.2*** | 0.4 | 24.6** | 6.2*** | -1.5 | 18.1* |
| no | 0.28 | 0.5 | 6.2*** | 0.4 | 24.5** | 6.2*** | -1.5 | 18.1* |
| no | 0.28 | 1 | 6.2*** | 0.4 | 30** | 6.2*** | -1.5 | 21.6** |
| no | 0.28 | 2 | 6.2*** | 0.4 | 39.6*** | 6.2*** | -1.5 | 28.9*** |
| no | 0.28 | 4 | 6.2*** | 0.4 | 37.8*** | 6.2*** | -1.5 | 28.2*** |
| no | 0.28 | Global | 6.2*** | 0.4 | 15.1** | 6.2*** | -1.5 | 14.7** |
| no | 0.28 | National | 6.2*** | 0.4 | 28.7** | 6.2*** | -1.5 | 21*** |
| no | 0.47 | 0.25 | 6.2*** | -3.6 | 20.6* | 6.2*** | -5.5* | 13.6 |
| no | 0.47 | 0.5 | 6.2*** | -3.6 | 20.5* | 6.2*** | -5.5* | 13.5 |
| no | 0.47 | 1 | 6.2*** | -3.6 | 26** | 6.2*** | -5.5* | 17.2* |
| no | 0.47 | 2 | 6.2*** | -3.6 | 35.6*** | 6.2*** | -5.5* | 25.6** |
| no | 0.47 | 4 | 6.2*** | -3.6 | 33.8*** | 6.2*** | -5.5* | 25.6** |
| no | 0.47 | Global | 6.2*** | -3.6 | 11.1* | 6.2*** | -5.5* | 10.2* |
| no | 0.47 | National | 6.2*** | -3.6 | 24.7** | 6.2*** | -5.5* | 17.8** |

Note: *p<0.1; **p<0.05; ***p<0.01

#### S5 Table: Regression Results for Pooled and Fixed Effects Specifications, Radius = 2.0


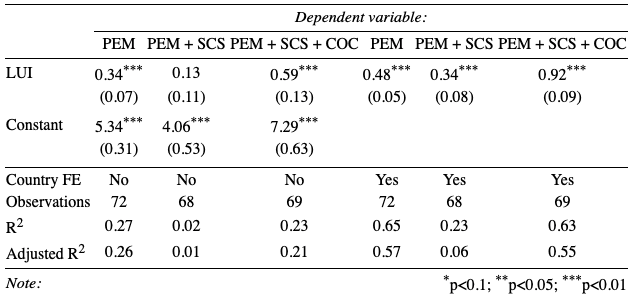


#### S6 Table: Beta Coefficient from Log-Log Regression of Carbon Footprint on Land Use Intensity with a Random Effect Specification Under Robustness Checks

| **COC set to zero for dry eco-climate zones?** | **SCS  (MgC ha^-1^ yr^-1^)** | **Radius/COC Type** | **Estimate** | **Adj R-Squared** | **n** |
| --- | --- | --- | --- | --- | --- |
| yes | 0.28 | 0.25 | 0.53*** | 0.26 | 69 |
| yes | 0.28 | 0.5 | 0.57*** | 0.27 | 69 |
| yes | 0.28 | 1 | 0.64*** | 0.4 | 69 |
| yes | 0.28 | 2 | 0.9*** | 0.63 | 69 |
| yes | 0.28 | 4 | 0.83*** | 0.63 | 69 |
| yes | 0.47 | 0.25 | 0.78*** | 0.42 | 63 |
| yes | 0.47 | 0.5 | 0.83*** | 0.43 | 63 |
| yes | 0.47 | 1 | 0.69*** | 0.38 | 65 |
| yes | 0.47 | 2 | 0.95*** | 0.59 | 66 |
| yes | 0.47 | 4 | 0.88*** | 0.59 | 66 |
| no | 0.28 | 0.25 | 0.61*** | 0.41 | 72 |
| no | 0.28 | 0.5 | 0.64*** | 0.42 | 72 |
| no | 0.28 | 1 | 0.68*** | 0.63 | 72 |
| no | 0.28 | 2 | 0.9*** | 0.87 | 72 |
| no | 0.28 | 4 | 0.85*** | 0.92 | 72 |
| no | 0.28 | Global | 0.75*** | 0.95 | 72 |
| no | 0.28 | National | 0.86*** | 0.98 | 72 |
| no | 0.47 | 0.25 | 0.79*** | 0.65 | 69 |
| no | 0.47 | 0.5 | 0.82*** | 0.65 | 69 |
| no | 0.47 | 1 | 0.68*** | 0.62 | 71 |
| no | 0.47 | 2 | 0.89*** | 0.83 | 72 |
| no | 0.47 | 4 | 0.83*** | 0.89 | 72 |
| no | 0.47 | Global | 0.72*** | 0.94 | 72 |
| no | 0.47 | National | 0.84*** | 0.98 | 72 |

Note: *p<0.1; **p<0.05;p<0.01

#### S7 Table: Studies Included in Regression Analysis

| **Study** | **Country** |
| --- | --- |
| Ridoutt et al. (2011) | Australia |
| Ridoutt et al. (2012) | Australia |
| Ridoutt et al. (2014) | Australia |
| Wiedemann et al. (2015b) | Australia |
| Wiedemann et al. (2016b) | Australia |
| Dick et al. (2015) | Brazil |
| Mazzetto et al. (2015) | Brazil |
| Schroeder et al. (2012) | Brazil |
| Siqueira and Duru (2016) | Brazil |
| Alemu et al. (2016) | Canada |
| Basarab et al. (2012) | Canada |
| Beauchemin et al. (2011) | Canada |
| Celis et al. (2013) | Chile |
| Veysset et al. (2011) | France |
| Widi et al. (2015) | Indonesia |
| Cederberg and Darelius (2000) | Sweden |
| Cederberg and Nilsson (2004b) | Sweden |
| Roop et al. (2013) | United States |
| Rotz et al. (2013) | United States |
| Jayasundara and Wagner-Riddle (2014) | Canada |
| Mc Geough et al. (2012) | Canada |
| Nguyen et al. (2013a) | France |
| Huerta et al. (2016) | Mexico |
| Roer et al. (2013) | Norway |
| Johansen et al. (2013) | Norway |
| Doublet et al. (2013b) | Romania |
| Ross et al. (2014) | United Kingdom |

#### S8 Table: Studies Included in Pasture-Finished (PF) / Grain-Finished (GF) Comparison

| **Pair** | **Study** | **Country** | **Geography** | **Type** | **Herd** |
| --- | --- | --- | --- | --- | --- |
| 1 | Wiedemann et al. (2015b) | Australia | New South Wales & Queensland | PF | Beef |
| 1 | Wiedemann et al. (2015b) | Australia | New South Wales & Queensland | GF | Beef |
| 2 | Wiedemann et al. (2015b) | Australia | New South Wales | GF | Beef |
| 2 | Wiedemann et al. (2016b) | Australia | New South Wales | PF | Beef |
| 3 | Cardoso et al. (2016) | Brazil | Cerrado | PF | Beef |
| 3 | Cardoso et al. (2016) | Brazil | Cerrado | GF | Beef |
| 4 | Pashei Kamali et al. (2016) | Brazil | South | PF | Beef |
| 4 | Pashei Kamali et al. (2016) | Brazil | South | GF | Beef |
| 5 | Celis et al. (2013) | Chile | Biobío | PF | Beef |
| 5 | Celis et al. (2013) | Chile | Biobío | GF | Beef |
| 6 | Mogensen et al. (2015) | Denmark | National | PF | Beef |
| 6 | Mogensen et al. (2015) | Denmark | National | GF | Beef |
| 7 | Mogensen et al. (2015) | Denmark | National | PF | Dairy |
| 7 | Mogensen et al. (2015) | Denmark | National | GF | Dairy |
| 8 | Nguyen et al. (2013a) | France | Normandy | PF | Dairy |
| 8 | Nguyen et al. (2013a) | France | Normandy | GF | Dairy |
| 9 | Casey and Holden (2006a); Blonk et al. (2008) | Ireland | National | GF | Beef |
| 9 | Casey and Holden (2006a); Blonk et al. (2008) | Ireland | National | PF | Beef |
| 10 | Huerta et al. (2016) | Mexico | North Centre, Veracruz | GF | Beef |
| 10 | Huerta et al. (2016) | Mexico | North Centre, Veracruz | PF | Beef |
| 11 | Cederberg and Darelius (2000) | Sweden | Halland county, [Götaland](https://en.wikipedia.org/wiki/G%C3%B6taland) | GF | Beef |
| 11 | Cederberg and Nilsson (2004b) | Sweden | Skâne county, [Götaland](https://en.wikipedia.org/wiki/G%C3%B6taland) | PF | Beef |
| 12 | Mogensen et al. (2015) | Sweden | National | PF | Dairy |
| 12 | Mogensen et al. (2015) | Sweden | National | GF | Dairy |
| 13 | Alig et al. (2012) | Switzerland | National | GF | Beef |
| 13 | Alig et al. (2012) | Switzerland | National | PF | Beef |
| 14 | Alig et al. (2012) | Switzerland | National | GF | Dairy |
| 14 | Alig et al. (2012) | Switzerland | National | PF | Dairy |
| 15 | Schroeder et al. (2012) | United Kingdom | Gloucestershire county | GF | Beef |
| 15 | Schroeder et al. (2012) | United Kingdom | Gloucestershire county | PF | Beef |
| 16 | Capper (2012) | United States of America | National | GF | Beef |
| 16 | Capper (2012) | United States of America | National | PF | Beef |
| 17 | Pelletier et al. (2010) | United States of America | Upper Midwest | GF | Beef |
| 17 | Pelletier et al. (2010) | United States of America | Upper Midwest | PF | Beef |
| 18 | Stanley et al. (2018) | United States of America | Midwest/Michigan | PF | Beef |
| 18 | Stanley et al. (2018) | United States of America | Midwest/Michigan | GF | Beef |
| 19 | Picasso et al. (2014) | Uruguay | North and East (Grassland) | PF | Beef |
| 19 | Picasso et al. (2014) | Uruguay | North and East/Rocha (Grassland) | GF | Beef |
| 20 | Picasso et al. (2014) | Uruguay | North and East (Pasture) | PF | Beef |
| 20 | Picasso et al. (2014) | Uruguay | North and East/Rocha (Pasture) | GF | Beef |
