# Supplementary material for: Carbon opportunity cost increases carbon footprint advantage of grain-finished beef
Source: PLoS One. 2023 Dec 13;18(12):e0295035. doi: 10.1371/journal.pone.0295035 (PMC10718409; doi:10.1371/journal.pone.0295035)
Supplement: Attachment — Submitted filename: PONE-D-22-23019_R2_reviewer.pdf [file pone.0295035.s003.pdf]

PLOS ONE

Carbon opportunity cost increases carbon footprint advantage of grain-finished beef

--Manuscript Draft--

**Manuscript Number:**	PONE-D-22-23019R2
**Article Type:**	Research Article
**Full Title:**	Carbon opportunity cost increases carbon footprint advantage of grain-finished beef
**Short Title:**	Carbon footprint of grain- and grass-finished beef
**Corresponding Author:**	Daniel Blaustein-Rejto, MPP<br>The Breakthrough Institute<br>Berkeley, California UNITED STATES
**Keywords:**	Land Use; climate change; agriculture; food systems; carbon footprint; beef; livestock; carbon opportunity cost; carbon sequestration; Climate mitigation
**Abstract:**	Beef production accounts for the largest share of global livestock greenhouse gas emissions and is an important target for climate mitigation efforts. Most life-cycle assessments comparing the carbon footprint of beef production systems have been limited to production emissions. None also consider potential carbon sequestration due to grazing and alternate uses of land used for production. We assess the total carbon footprint of 100 beef production systems in 16 countries, including production emissions, soil carbon sequestration from grazing, and carbon opportunity cost—the potential carbon sequestration that could occur on land if it were not used for production. We conduct a pairwise comparison of pasture-finished operations in which cattle almost exclusively consume grasses and forage, and grain-finished operations in which cattle are first grazed and then fed a grain-based diet. We find that pasture-finished operations have 20% higher production emissions and 42% higher total carbon footprint than grain-finished systems. We also find that more land-intensive operations generally have higher carbon footprints. Regression analysis indicates that a 10% increase in land-use intensity is associated with a 4.8% increase in production emissions, but a 9.0% increase in the total carbon footprint, including production emissions, soil carbon sequestration and carbon opportunity cost. The carbon opportunity cost of operations was, on average, 130% larger than production emissions. These results point to the importance of accounting for carbon opportunity cost in assessing the sustainability of beef production systems and developing climate mitigation strategies.
**Order of Authors:**	Dan Blaustein-Rejto, MPP
	Linus Blomqvist, MESc
	Nicole Soltis, PhD
**Response to Reviewers:**	As suggested by PLOS ONE editorial staff, we have addressed the reviewer comments regarding S1 Fig in the Supplementary Information by ensuring it follows journal guidelines. We confirmed that the basemap of the figure is in the public domain. It therefore meets PLOS ONE's licenses and copyright policy for figures. A statement from Natural Earth providing proof that the material is in the public domain is attached as "other file" and can be viewed on their website here: https://www.naturalearthdata.com/about/terms-of-use/.<br><br>As there were no additional reviewer comments, the manuscript, supplementary information, and figures have not been modified. Since the PLOS ONE Editorial Manager requires authors to attach a revised manuscript with and without track changes, we have attached the same manuscript and supplementary information files as we did when originally responding to reviewer comments. We apologize for any confusion this causes.
**Additional Information:**	
**Question**	**Response**
**Financial Disclosure**	The authors received no specific funding for this work.

Powered by Editorial Manager® and ProduXion Manager® from Aries Systems Corporation

Enter a financial disclosure statement that describes the sources of funding for the work included in this submission. Review the submission guidelines for detailed requirements. View published research articles from *PLOS ONE* for specific examples.<br><br>This statement is required for submission and **will appear in the published article** if the submission is accepted. Please make sure it is accurate.<br><br>**Unfunded studies**<br>Enter: *The author(s) received no specific funding for this work.*<br><br>**Funded studies**<br>Enter a statement with the following details:<br>• Initials of the authors who received each award<br>• Grant numbers awarded to each author<br>• The full name of each funder<br>• URL of each funder website<br>• Did the sponsors or funders play any role in the study design, data collection and analysis, decision to publish, or preparation of the manuscript?<br>• **NO** - Include this sentence at the end of your statement: *The funders had no role in study design, data collection and analysis, decision to publish, or preparation of the manuscript.*<br>• **YES** - Specify the role(s) played.<br><br>* typeset	
**Competing Interests**<br><br>Use the instructions below to enter a competing interest statement for this submission. On behalf of all authors, disclose any competing interests that could be perceived to bias this work—acknowledging all financial support and any other relevant financial or non-financial competing interests.<br><br>This statement is required for submission and **will appear in the published article** if	The authors have declared that no competing interests exist.

the submission is accepted. Please make sure it is accurate and that any funding sources listed in your Funding Information later in the submission form are also declared in your Financial Disclosure statement.<br><br>View published research articles from *PLOS ONE* for specific examples.<br><br>**NO authors have competing interests**<br><br>Enter: *The authors have declared that no competing interests exist.*<br><br>**Authors with competing interests**<br><br>Enter competing interest details beginning with this statement:<br><br>*I have read the journal's policy and the authors of this manuscript have the following competing interests: [insert competing interests here]*<br><br>* typeset	
**Ethics Statement**<br><br>Enter an ethics statement for this submission. This statement is required if the study involved:<br><br>• Human participants<br>• Human specimens or tissue<br>• Vertebrate animals or cephalopods<br>• Vertebrate embryos or tissues<br>• Field research<br><br>Write "N/A" if the submission does not require an ethics statement.<br><br>General guidance is provided below. Consult the submission guidelines for detailed instructions. **Make sure that all information entered here is included in the Methods section of the manuscript.**	N/A

**Format for specific study types**<br><br>**Human Subject Research (involving human participants and/or tissue)**<br>• Give the name of the institutional review board or ethics committee that approved the study<br>• Include the approval number and/or a statement indicating approval of this research<br>• Indicate the form of consent obtained (written/oral) or the reason that consent was not obtained (e.g. the data were analyzed anonymously)<br><br>**Animal Research (involving vertebrate animals, embryos or tissues)**<br>• Provide the name of the Institutional Animal Care and Use Committee (IACUC) or other relevant ethics board that reviewed the study protocol, and indicate whether they approved this research or granted a formal waiver of ethical approval<br>• Include an approval number if one was obtained<br>• If the study involved *non-human primates*, add *additional details* about animal welfare and steps taken to ameliorate suffering<br>• If anesthesia, euthanasia, or any kind of animal sacrifice is part of the study, include briefly which substances and/or methods were applied<br><br>**Field Research**<br><br>Include the following details if this study involves the collection of plant, animal, or other materials from a natural setting:<br>• Field permit number<br>• Name of the institution or relevant body that granted permission	
**Data Availability**<br><br>Authors are required to make all data underlying the findings described fully available, without restriction, and from the time of publication. PLOS allows rare exceptions to address legal and ethical concerns. See the PLOS Data Policy and FAQ for detailed information.	Yes - all data are fully available without restriction

A Data Availability Statement describing where the data can be found is required at submission. Your answers to this question constitute the Data Availability Statement and **will be published in the article**, if accepted.<br><br>**Important:** Stating 'data available on request from the author' is not sufficient. If your data are only available upon request, select 'No' for the first question and explain your exceptional situation in the text box.<br><br>Do the authors confirm that all data underlying the findings described in their manuscript are fully available without restriction?	
**Describe where the data may be found in full sentences. If you are copying our sample text, replace any instances of XXX with the appropriate details.**<br><br>• If the data are **held or will be held in a public repository**, include URLs, accession numbers or DOIs. If this information will only be available after acceptance, indicate this by ticking the box below. For example: *All XXX files are available from the XXX database (accession number(s) XXX, XXX.*).<br>• If the data are all contained **within the manuscript and/or Supporting Information files**, enter the following: *All relevant data are within the manuscript and its Supporting Information files.*<br>• If neither of these applies but you are able to provide **details of access elsewhere**, with or without limitations, please do so. For example:<br><br>*Data cannot be shared publicly because of [XXX]. Data are available from the XXX Institutional Data Access / Ethics Committee (contact via XXX) for researchers who meet the criteria for access to confidential data.*<br><br>*The data underlying the results presented in the study are available from (include the name of the third party*	All relevant data are within the manuscript and its Supporting Information files.

and contact information or URL).<br>• This text is appropriate if the data are owned by a third party and authors do not have permission to share the data.<br><br>* typeset	
Additional data availability information:	

Powered by Editorial Manager® and ProduXion Manager® from Aries Systems Corporation

Manuscript

Click here to access/download;Manuscript;manuscript_clean.docx

Carbon opportunity cost increases carbon footprint advantage of grain-finished beef

Dan Blaustein-Rejto[1*], Linus Blomqvist[1,2], Nicole Soltis[3]

[1] The Breakthrough Institute, Berkeley, California, United States of America

[2] Bren School of Environmental Science & Management, University of California, Santa Barbara, California, United States of America

[3] California Council on Science and Technology, Sacramento, California, United States of America

*Corresponding author

Email: dan@thebreakthrough.org (DBR)

Abstract

Beef production accounts for the largest share of global livestock greenhouse gas emissions and is an important target for climate mitigation efforts. Most life-cycle assessments comparing the carbon footprint of beef production systems have been limited to production emissions. None also consider potential carbon sequestration due to grazing and alternate uses of land used for production. We assess the total carbon footprint of 100 beef production systems in 16 countries, including production emissions, soil carbon sequestration from grazing, and carbon opportunity cost—the potential carbon sequestration that could occur on land if it were not used for production. We conduct a pairwise comparison of pasture-finished operations in which cattle almost exclusively consume grasses and forage, and grain-finished operations in which cattle are first grazed and then fed a grain-based diet. We find that pasture-finished operations have 20% higher production emissions and 42% higher total carbon footprint than grain-finished systems. We also find that more land-intensive operations generally have higher carbon footprints. Regression analysis indicates that a 10% increase in land-use intensity is associated with a 4.8% increase in production emissions, but a 9.0% increase in the total carbon footprint, including production emissions, soil carbon sequestration and carbon opportunity cost. The carbon opportunity cost of operations was, on average, 130% larger than production emissions. These results point to the importance of accounting for carbon opportunity cost in assessing the sustainability of beef production systems and developing climate mitigation strategies.

Introduction

Beef production accounts for about 6% of all anthropogenic ~~greenhouse gas emissions~~ [1]. Given rising demand in developing countries, reducing the greenhouse-gas (or carbon) footprint

of production, measured as kilograms carbon dioxide-equivalent (CO_2e) per kilogram of beef, is an important climate mitigation strategy [2-3].

Whether beef is produced in pasture-finished or grain-finished systems affects its carbon footprint. In both pasture-finished and grain-finished systems, cattle are raised initially on pasture or rangeland. The primary difference lies in the finishing stage—in grain-finished systems, cattle are fed a grain-based diet and often kept in feedlots, whereas cattle in pasture-finished systems continue to eat fresh and stored grasses and hay until they reach slaughter weight [4]. The finishing stage therefore accounts for any potential difference in the carbon footprint of these systems. Pasture-finished systems are common in many parts of the world and account for approximately 33% of global beef production. Grain-finished systems account for 15%, and other systems, such as mixed crop-livestock production, account for the remainder [5].

Most life-cycle assessments of the carbon footprint of grain-finished and pasture-finished systems have been limited to emissions directly attributable to cradle-to-farmgate activities (here referred to as production emissions) [6]. Reviews and meta-analyses of these studies conclude that pasture-finished systems have a higher average carbon footprint [4,6,7]. Grain finishing typically leads to much higher growth rates. As a result, proportionally less energy is expended on maintenance rather than growth, such that inputs and emissions per unit of beef is lower [8].

In addition to emissions associated with production, beef's carbon footprint is also influenced by land use. Recent meta-analyses show that pasture-finished systems have higher land-use intensity (measured as area per unit production) on average, since the amount of pasture needed in the finishing stage of pasture-finished cattle is much larger than the amount of cropland needed to provide grain for the finishing stage of grain-finished cattle [46].

Greater land requirements influence the carbon footprint in two ways. First, pasture and crop management can increase soil carbon sequestration [9-10]. Use of improved grazing practices in some pasture-finished systems has sequestered enough carbon to offset production emissions from finishing [11]. Yet large soil carbon sequestration rates are only possible under particular agro-ecological conditions and for a limited time period [9,12].

Second, greater land use for beef production can displace native ecosystems and reduce land available for restoration. The amount of CO_2 that could be removed on land used for production through reforestation or other restoration has been referred to as the "carbon opportunity cost" [13].

Existing global comparisons of pasture-finished and grain-finished systems are incomplete as they do not account for both carbon opportunity cost and soil carbon sequestration. For instance, Poore and Nemecek (2018) [6], in a global meta-analysis of life-cycle assessments, do not account for potential soil carbon sequestration from production or the carbon opportunity cost of land use. The authors do account for emissions from land-use change, but only from recent changes in which total area for the crop or livestock product increased in the country of production. This approach, unlike the carbon opportunity cost approach, can result in zero carbon costs associated with many types of land use (see Searchinger *et al.* 2018 [14] Supplementary Discussion for a detailed treatment). Balmford *et al.* (2018) [15] estimate the relationship between the carbon footprint and land-use intensity of beef production including foregone carbon sequestration from land use—finding that there is a strong positive correlation—but their analysis is limited to Latin America and does not estimate soil carbon sequestration from grazing. Schmidinger and Stehfest (2012) [16], Searchinger *et al.* (2018) [14], and Hayek *et al.* (2020) [13] estimate the carbon opportunity cost of beef production at different geographic

scales, but do not compare grain-finished and pasture-finished systems or estimate soil carbon sequestration from grazing.

Here, for the first time, we assess the total carbon footprint – defined as the sum of carbon emissions from production, soil carbon sequestration, and carbon opportunity cost – of pasture-finished and grain-finished systems from across the world. We compare the total carbon footprint of pasture-finished and grain-finished systems that exist in the same region and that have been studied using the same methodology. To assess the relationship between land-use intensity and carbon footprint, regardless of the system, we also regress several carbon footprint measures on land-use intensity.

Beef production systems are changing rapidly across the world, and decisions about the future direction of this change will have important implications for climate mitigation as well as other environmental impacts. Accounting for the total carbon footprint, including the carbon opportunity cost, as we do in this paper, should help guide these decisions.

Materials and methods

We calculate the total carbon footprint (the sum of production emissions, soil carbon sequestration, and carbon opportunity costs in kilograms CO_2e per kilogram of retail weight beef) of 100 beef production operations across 16 countries, including those from beef and dairy herds, drawn from a dataset of food and beverage life-cycle assessments [9] and from Stanley *et al.* (2018) [11]. Poore and Nemecek (2018) [9] includes production emissions and land-use intensity data. Stanley *et al.* (2018) [11] reports production emissions, carbon sequestration, emissions from soil erosion, and land-use intensity for the finishing stage of a pasture-finished and grain-finished operation in the Midwestern USA; we derive values from earlier stages from

Pelletier *et al.* (2010) [17] which also studied operations in the Midwest. We conduct a pair-wise comparison of carbon footprints between pasture-finished and grain-finished beef production systems, and a regression analysis of the relationship between land-use intensity and carbon footprint.

Production emissions and land-use intensity

Production emissions represent cradle-to-farmgate life-cycle greenhouse gas emissions. This includes emissions associated with enteric fermentation, animal housing, manure management, and inputs associated with feed production such as fertilizers, pesticides, and machinery.

Land-use intensity represents land required for grazing and crop production, in hectare per kilogram of retail weight beef. Land use for pasture is calculated as the sum of temporary and permanent pasture, and land use for cropland is calculated as the sum of seed, arable and fallowed crop land. We use and standardize production emissions and land-use intensity values from Poore and Nemecek (2018) [9] and Stanley *et al.* (2018) [11].

Soil carbon sequestration

Soil carbon sequestration (SCS) in kg CO_2 per kg of retail weight beef is calculated as the product of land-use intensity of grazing (LUI) and carbon sequestration due to grazing (CSG) in kg C ha^{-1} yr^{-1} (Equation 1).

$$SCS = LUI \cdot CS \cdot \frac{44\ CO_2}{12\ C} \qquad (1)$$

There is insufficient data to calculate a specific carbon sequestration rate for each life-cycle assessment location. This is in part because sequestration rates depend on environmental and management factors, such as soil texture and grazing intensity, not consistently described in the life-cycle assessments. Instead, for all life-cycle assessments we use the mean carbon

sequestration rate of 0.28 Mg C ha^{-1} yr^{-1} for "improved grazing management" estimated in a synthesis of the grassland management literature [18]. This estimate, drawn from studies with an average soil depth of 23 cm, is within the range of peer reviewed estimates: 0.03 and 1.04 Mg C $ha^{-1}yr^{-1}$, with the lowest values corresponding to dry climates and the highest to specific grassland management practices and regions [19]. Given that not all the life-cycle assessments included are of operations with improved grazing practices, the true carbon sequestration rates across operations may be lower. To be conservative in our carbon footprint for grain-finished operations, we assume that no carbon sequestration occurs on cropland used for feed production, consistent with research that shows that CO_2 emissions from agricultural land are generally balanced by removals [20].

Carbon opportunity cost

Our measure of carbon opportunity cost calculates how much carbon sequestration would have occurred had land been occupied with native ecosystems instead of pasture or cropland. This assumes that reducing land-use intensity results in proportionately less agricultural land area locally.

We calculate carbon opportunity cost (COC) as the sum of the carbon opportunity cost of pasture (p) and cropland (c) used in production. For each of these two land uses, the carbon opportunity cost is calculated as the product of land-use intensity (LUI) and potential carbon sequestration (PCS) of the land in the area where the life-cycle assessments was conducted, in kg C ha^{-1} yr^{-1} (Equations 2 and 3).

$$COC = \sum_i LUI_i \cdot PCS_i \cdot \frac{44\ CO_2}{12\ C} \text{ for } i = c, p \qquad (2)$$

where

$$PCS_i = \frac{NPP_i \cdot k_i \cdot r - s_i}{r} \text{ for } i = c, p \qquad (3)$$

NPP_i denotes the potential net primary productivity of native vegetation (kg C ha^{-1} yr^{-1}) that could be restored on agricultural land within a given radius of where the life-cycle assessment was conducted. We report results using a radius of 2 degrees (~223 km at equator). k_i is the conversion factor from net primary productivity to carbon sequestration in vegetation and soils or, put differently, the average level of carbon sequestration generated by devoting one kilogram of NPP to restoring native vegetation. This value is 0.42 kg CO_2 ha^{-1} yr^{-1} for every kg of NPP for cropland and 0.44 for pasture, as calculated by Searchinger *et al.* (2018) [14]. r denotes the time period over which carbon sequestration is averaged, in this case 100 years; and s_i denotes existing vegetation carbon stocks (kg C ha^{-1}), 1100 for cropland and 3100 for pasture, based on global averages for cereals and pasture, respectively, from Searchinger *et al.* (2018) [14]. Although spatially explicit estimates of cropland carbon stocks exist [21], we are not aware of any for pasture carbon stocks.

The logic behind Equation 3 is as follows. The numerator represents the difference in potential carbon stocks between current land use and native vegetation. $NPP_i \cdot k_i$ is a flux measure, in kilograms of carbon per hectare per year, which we multiply by 100 to turn into a stock measure. In effect, this assumes that the equilibrium carbon stock in native ecosystem is reached after 100 years. The numerator, the difference in potential carbon stocks, is then divided by 100 to arrive at an annual (flux) rate. We select a time period of 100 years based on previous studies such as Searchinger *et al.* (2018) [14] and Schmidinger and Stehfest (2012) [16], which use it as a time period over which to calculate average carbon sequestration rates in regenerating forests.

Data on potential net primary productivity under native vegetation is generated by the Lund–Potsdam–Jena managed Land (LPJmL) model, a dynamic global vegetation model that simulates vegetation composition, distribution, and carbon stocks and flows at 0.5x0.5° spatial resolution. We use LPJmL results from Searchinger *et al.* (2018) [14].

We assume life-cycle assessment sites located in climate categorized as "dry" in Poore & Nemecek (2018) [9] have zero potential carbon sequestration because they either cannot support substantial additional biomass or are native grasslands or savannas for which restoration does not typically involve reforestation [22].

Pairwise comparison between pasture-finished and grain-finished production systems

We compare the carbon footprint of 20 pairs of pasture-finished and grain-finished production systems, across 12 countries, in the Poore and Nemecek (2018) [9] database and one recent comparative life-cycle assessment [11] with and without soil carbon sequestration and carbon opportunity cost included. Systems were selected for inclusion if they were in the same subnational region or country, if the study was national in scope, and reported in the same study or within two studies by the same primary author. Details of the pairs are listed in S8 Table. Fourteen of the pairs were reported for the same geographic region, but lacked coordinates. For those, we estimated carbon opportunity cost by calculating mean potential net primary productivity on cropland and grazing land within the subnational region or country the life-cycle assessment was located (Supplementary Methods). We used a paired t-test to test if the mean difference between the pasture-finished and grain-finished system was significantly different from zero.

Regression analysis

We also assess the relationship between carbon footprint and land-use intensity using cross-section regression analysis of beef production operations. We include 72 operations from life-cycle assessments that report geographic coordinates, including a total of 24 studies in 12 countries (S1 Fig, S7 Table). We log-transform the carbon footprint and land-use intensity because the input data is heavily right-skewed and because this enables us to present results as elasticities—the expected percent change in the carbon footprint with a percentage change in land-use intensity.

We run three different regressions, starting with production emissions as the only regressor, adding carbon opportunity cost in the second regression, and then also including soil carbon sequestration in the third regression. We use a linear model to facilitate comparison of the relationship across the regressions. Since there may be variables operating at the country level that influence the carbon footprint (e.g. climate, national policy), we use a multilevel model with country-level random effects, particularly varying intercepts and constant slopes (Gelman and Hill, 2007). This yields the following regression equation:

$$log(carbon\ footprint_{i,j}) = \beta_0 + \beta_1 log(LUI_{i,j}) + u_j + \epsilon_{i,j} \qquad (4)$$

where j indexes countries, i indexes operations within countries, $\beta_0 + u_j$ is the intercept for each country, β_1 represents the elasticity between land-use intensity and the carbon footprint, and ϵ_{ij} is an error term.

We choose this specification over a fixed effect model as there is substantial variation in the independent variable within units (i.e. countries), the level of correlation between unit effects and the independent variable is not extremely high, and we are interested in accounting for the variability between units but not in estimating specific unit effects, in which case a random

effects model can be appropriate to use and result in superior estimates (Clark and Linzer 2015). Regressions with fixed effects produced results very similar to those with random effects (S5 Table S5). Our analysis examines differences in total carbon footprint across operations with different land-use intensity and does not attempt causal inference per se.

Robustness checks

We vary four parameters to assess the robustness of the results. First, we run the analysis with 0.25, 0.5, 1.0 and 4 degree radius. We do this to confirm our results cannot be explained by the choice of radius as NPP values can vary widely over a small area.

Second, we run the analysis with alternative calculations for carbon opportunity cost at the national and global levels. The national and global carbon opportunity costs assume that if the amount of land needed to support a given level of food production declines by one unit as a result of lower land-use intensity, then one unit of land will be restored to native vegetation somewhere in the country or world, respectively. These are relevant comparisons in cases where domestic and international trade allow land-use intensity reductions to be spatially disconnected from pasture and cropland expansion/contraction. We calculate national carbon opportunity cost using the average NPP values over all crop and pasture land across the country each production system is located in. This method could be improved by using crop-specific values; however, not all life-cycle assessments in our dataset describe which crops are used in production. We also calculate global carbon opportunity cost using average global net primary production values.

Third, we run the analysis using a carbon sequestration rate of 0.47 Mg C ha^{-1} yr^{-1}, the average value reported across all studies of improved grassland management included in Conant *et al.* (2017). This reduces the total carbon footprint of more land-intensive operations such as

pasture-finished systems more than it reduces the carbon footprint of less land-intensive operations.

Fourth, we run the analysis with and without the potential carbon sequestration, and thus the carbon opportunity cost, set to 0 for operations in dry climates.

Results

In this study we calculated the total carbon footprint of beef production systems as the sum of production emissions, carbon opportunity cost, and soil carbon sequestration, and assessed the relationship of total carbon footprint and land-use intensity. After presenting summary statistics, we show the results of the pair-wise comparison of the carbon footprints of pasture-finished and grain-finished beef production systems. We then present results from regression analysis of carbon footprints on land-use intensity, with separate regressions for the different approaches for calculating carbon footprint.

The total carbon footprint across the 72 beef production operations with reported latitude and longitude, and the 28 operations without latitude/longitude included in the pasture-finished/grain-finished comparison ranged from -68.3 to 2169.3 kg CO_2e kg^{-1}retail weight, with mean 177.37 and median 107.14 (Table 1). Four pasture-finished and one grain-finished production systems in Queensland, Australia are estimated to have negative carbon footprints, in part because we assume that the dry climate results in zero carbon opportunity cost. If soil carbon sequestration rates are lower in dry climates than other climates, as some studies such as Smith *et al.* (2008) suggest, these operations would be more likely to also have positive carbon footprints. The total carbon footprint was similar in robustness checks, with the mean value

ranging from 141.6 to 210.0 kg CO_2e kg^{-1} retail weight when different radii are used and when we do not assume zero carbon opportunity cost for arid climates (S1 Table).

Table 1: Summary statistics for beef operations

Variable	Mean	Median	Range	95% CI	Units
Production emissions	52.64	41.42	4.9, 182	45.48, 59.8	kg CO_2e kg^{-1}
Soil carbon sequestration	-15.11	-7.41	-164.8, 0	-19.96, -10.26	kg CO_2e kg^{-1}
Carbon opportunity cost	139.85	68.46	0, 2243	87.1, 192.59	kg CO_2e kg^{-1}
Total carbon footprint	177.37	107.14	-68.3, 2169.3	124.79, 229.96	kg CO_2e kg^{-1}
Land-use intensity	0.02	0.01	0, 0.2	0.01, 0.02	ha kg^{-1}

All units are per kilogram retail weight. $n = 100$.

In individual systems, carbon opportunity cost was, on average, 130% larger than production emissions. Soil carbon sequestration offset 31.5% of production emissions and 18.9% of the production emissions and carbon opportunity cost, on average. Across all robustness checks, carbon opportunity cost is at least 65% larger than production emissions and soil carbon sequestration does not fully offset production emissions (S2 Table).

Pairwise comparison between pasture-finished and grain-finished systems

The pairwise comparison found that pasture-finished systems had 20% higher mean production emissions than grain-finished systems on average ($p<0.01$). When also including soil carbon sequestration, the difference is not statistically significant at a 95% confidence level ($p\geq0.05$). When the carbon opportunity cost is also accounted for, however, the total carbon footprint of pasture-finished systems is on average 42% higher than that of grain-finished systems ($p<0.01$) (Fig 1). Compared to grain-finished systems, pasture-finished systems also had 15% higher median production emissions ($p<0.01$) and total carbon footprints ($p<0.05$), indicating that while the magnitude of the difference is sensitive to extreme values, the general finding of higher emissions is robust (S3 Table).

Fig 1: Average ratios of carbon footprints between pasture-finished and grain-finished. Ratios expressed as percentage difference. PEM denotes production emissions, SCS denotes soil carbon sequestration, and COC denotes carbon opportunity cost. Values above (below) 0 denote the carbon footprint for pasture-finished operations is larger (smaller) than for grain-finished operations. Comparisons were made within paired production systems to control for agronomic and environmental differences. Bars show means and 95% confidence intervals. On average, carbon footprints for pasture-finished operations are significantly greater ($p<0.01$) than those of grain-finished operations when only production emissions are included and when production emissions, soil carbon sequestration and carbon opportunity cost are included. *n* = 20 pairs.

The carbon footprint of pasture-finished systems, including production emissions, soil carbon sequestration and carbon opportunity cost, is higher than that of the grain-finished systems ($p<0.05$) in the majority of robustness tests (S4 Table). Differences are not significant ($p\geq0.05$) in some cases when a smaller radius or higher rate of soil carbon sequestration is used.

Regression analysis

In the regression analysis, when only production emissions are regressed on land-use intensity, the coefficient is 0.48 (Fig 2a, Table 2). This can be interpreted as a 10% increase in land-use intensity being associated with a 4.8% increase in carbon footprint. Less land-intensive systems typically have lower carbon footprints, measured by production emissions alone. Fig 2a shows the regression line with this slope, with the level adjusted by country. When adding in soil carbon sequestration, the coefficient is reduced to 0.32, indicating that soil carbon sequestration offsets a part of the production emissions (Table 2).

Fig 2: The relationship between land-use intensity and carbon footprint of beef production systems. Results from a regression of log(carbon footprint) on log(land-use intensity) with country random effects. Dots indicate life-cycle assessment observations; colors indicate countries; and lines represent the slope of the regression that includes all countries, adjusted according to the levels of each country. A) Carbon footprint including only production emissions. n = 72. B) Carbon footprint including production emissions, soil carbon sequestration and carbon opportunity cost. n = 69.

Table 2: Results from log-log regressions

	Dependent variable:		
	PEM	PEM+SCS	PEM+SCS+COC
LUI	0.48***	0.32***	0.90***
	(0.04)	(0.08)	(0.09)
Constant	5.90***	4.84***	8.70***
	(0.27)	(0.45)	(0.52)
Observations	72	68	69
R^2	0.67	0.27	0.63
Adjusted R^2	0.66	0.25	0.63

Standard errors in parentheses. LUI = land-use intensity. PEM = production emissions. SCS = soil carbon sequestration. COC = carbon opportunity cost.
* $p < 0.1$, ** $p < 0.05$, *** $p < 0.01$

However, the relationship between total carbon footprint, including carbon opportunity cost, and land-use intensity is stronger, with a coefficient of 0.90 (Table 2, Fig 2b). Hence, a 10% increase in land-use intensity is associated with a 9.0% increase in the total carbon footprint of beef production. This near-proportional relationship is in part due to the large share of the total carbon footprint accounted for by carbon opportunity cost, which is proportional to land area in production.

Regressions with pooled and country fixed-effects specifications generate similar results (S5 Table). Results are robust to other specifications and assumptions checked (S6 Table).

Discussion

Our analysis is the first global comparison of the carbon footprint of grain-finished and pasture-finished beef production systems that includes production emissions as well as soil

carbon sequestration and carbon opportunity cost. This yields significant new insights that can inform environmental and agricultural decision-making.

Our results indicate that pasture-finished and other more land-intensive beef production systems have greater production emissions than grain-finished and less land-intensive systems. When we calculate carbon footprints including production emissions, soil carbon sequestration, and carbon opportunity cost, all beef production systems have a higher carbon footprint than when only production emissions are included, but pasture-finished systems have a substantially larger carbon footprint than grain-finished systems, and there is a strong positive relationship between land use intensity and carbon footprint.

The differences in carbon footprint between pasture- and grain-finished operations are largely due to differences in carbon opportunity cost, which account for a large share of the total carbon footprint. The carbon opportunity cost of operations was, on average, 130% larger than production emissions. These results point to the importance of accounting for carbon opportunity cost in assessing the sustainability of beef production systems.

Our analysis also confirms that beef operations that have been studied in life-cycle assessments are generally not carbon neutral or negative. The mean carbon footprint across all studies, including production emissions, sequestration, and carbon opportunity cost, is over three times larger than the mean value for production emissions (Table 1). One exception is that we estimate negative carbon footprints for several grass-finished operations and one grain-finished operation that are in dry eco-climate zones in Australia, for which we assume there is zero carbon opportunity cost. This suggests that grazing cattle on dry rangeland with little to no carbon opportunity cost could have a small carbon footprint when the grazing also increases soil

organic carbon, as has been observed in some studies of dry rangeland with finer textured soil [12].

Our comparison of pasture-finished and grain-finished systems builds upon and strengthens past findings. Our finding that production emissions are 20% higher on pasture-finished operations than on grain-finished operations is consistent with Clark and Tilman (2017) [6], which found average emissions were 19% higher though their estimate was not statistically significant. In our results, soil carbon sequestration from grazing offsets only a portion of production emissions. This finding is consistent with the conclusions of Garnett *et al.* (2017) [19], which estimated that soil carbon sequestration from grazing can offset 20-60% of annual emissions from ruminant grazing.

Our finding that land-use intensity and carbon footprint are positively correlated strengthens similar findings from previous studies, none of which included production emissions, soil carbon sequestration and carbon opportunity cost, which is a more comprehensive approach for assessing the carbon footprint of land use than conventional land-use change approaches [16]. Poore and Nemecek (2018) [9] found that beef and lamb systems with lower land-use intensity have a lower carbon footprint when considering land-use change-related greenhouse-gas emissions, but not carbon opportunity cost. Balmford *et al.* (2018) [14] used generalized linear mixed models to analyze the relationship between land-use intensity and carbon footprint, including a measure of carbon opportunity cost based on IPCC (2006) methods. Their analysis, limited to Brazil and tropical Mexico, also found that the carbon opportunity cost of agriculture was typically greater than production emissions, and that incorporating opportunity costs generated strongly positive associations between carbon footprint and land-use intensity. Searchinger *et al.* (2018) [14] calculated global-average carbon opportunity costs for beef similar

to the average calculated for all operations included in this study. Their estimates of 165.3 and 143.9 kg CO_2e kg^{-1} carcass weight were based on the potential carbon that could be gained or lost, respectively, on land used for production. The authors applied the values to five production systems in Brazil and found, consistent with our results, that systems with the lowest land-use intensity had the greatest carbon benefits.

Our study has several limitations although we do not believe these substantially alter our conclusions. The pairwise comparison of grain-finished and pasture-finished operations has a relatively small sample of 20 pairs. This means that assumptions of asymptotic normality, which are the basis for the paired t-test, may not hold. However, our robustness checks (S4 Table) and nonparametric test of the median (S3 Table), which is robust to small sample sizes, extreme outliers, and heavy-tailed distributions, reinforce the conclusion that pasture-finished operations have greater production emissions and total carbon footprints than grain-finished operations. In addition, our results cannot be considered to be globally representative or representative of all operations. The life-cycle assessments that underlie our study were not conducted to be globally representative. For instance, we include one study from Asia (Indonesia) and none from Africa.

In our study, we assume that a change in land-use intensity results in a proportionate change in land under production and thus the land area with native ecosystems. While this has the advantage of simplicity, it is unlikely to be exactly true in reality, as a result of economic mechanisms. The real effect may be more or less than proportional depending, in part, on how differences in land-use intensity and carbon footprint are associated with total factor productivity. For instance, an operation shifting from grain-finished to pasture-finished may lower total factor productivity. This would increase prices and lead to a reduction in overall demand, while at the same time making that operation less profitable and thus induce producers

elsewhere to produce more. The reduction in demand would reduce land use and the spillover of production would increase land use, with an ambiguous net impact.

It is also challenging to predict where a change in farmland area and native vegetation will take place as a result of changes in land-use intensity and production system in a given location. We calculate three measures of carbon opportunity cost: local, national, and global. These roughly correspond to different levels of market connectedness, which will differ between locations. For example, changes in US production can have large effects on global markets, whereas changes in less globally connected regions such as sub-Saharan Africa will likely see mostly local or national effects [24]. Furthermore, for those producers connected to global markets, effects of changes in production are not likely to be evenly distributed across the world, but are likely to be concentrated in those regions that are more globally integrated [24]. In the last few decades, much of the expansion of pasture has taken place in tropical countries like Brazil [25]. Following this logic, it is possible that higher land-use intensity in the US as a result of shifting to pasture-finished systems would displace production to these places, and is thus more likely to displace highly carbon-rich tropical ecosystems.

In addition, we use several simplifying assumptions. We use global mean estimates of soil carbon sequestration and current carbon stocks in cropland and grazing land vegetation due to lack of spatially-explicit data with global coverage. Our assumed rate is drawn from estimates for improved grazing management, so as to lessen the risk of overestimating the carbon footprint of grass-finished systems. Our measures of carbon opportunity cost are also based on mean potential carbon sequestration values in grazing land and cropland, if restored to native vegetation. They do not account for livestock diet rations, which crops are used for feed, or crop yields for instance. This may contribute to us underestimating potential carbon sequestration and

carbon opportunity costs if feed crops such as soy are grown in areas with higher potential carbon sequestration, such as former forest, than other crops.

Future research could build upon our analysis by integrating more spatially explicit estimates of soil carbon sequestration and carbon stocks and calculating carbon opportunity cost based on how different cropland and grazing land is used in beef production. Further types of beef and other livestock operations, such as pork or milk, could also be studied with similar methods.

Overall, this study provides a novel assessment of the carbon footprint of beef operations, building upon life-cycle assessments of production emissions to also include carbon sequestration and carbon opportunity cost. Our conclusion that beef operations with low land-use intensity, including grain-finished operations, have lower total carbon footprints than pasture-finished operations and others with high land-use intensity provides important insights for agricultural stakeholders. Accounting for products' total carbon footprint, not just production emissions, could shift which production systems government programs, corporate procurement, investors, and consumers incentivize.

Acknowledgements

The authors would like to thank Kenton de Kirby, Ken Cassman, and Joseph Poore for valuable comments on the draft manuscript.

References

1. Steinfeld H, Gerber P, Wassenaar T D, Castel V, Rosales M, Rosales M and de Haan C. Livestock's long shadow: Environmental issues and options. 2006. Food and Agriculture Organization of the United Nations. Available at: https://www.fao.org/3/a0701e/a0701e.pdf
2. Clark M A, Domingo N G G, Colgan K, Thakrar S K, Tilman D, Lynch J, et al. Global food system emissions could preclude achieving the 1.5° and 2°C climate change targets. Science. 2020;370: 705–8. doi: 10.1126/science.aba7357
3. Swain M, Blomqvist L, McNamara J and Ripple W J. Reducing the environmental impact of global diets. Sci. Total Environ. 2018: 1207–9. doi: 10.1016/j.scitotenv.2017.08.125
4. Clark M and Tilman D. Comparative analysis of environmental impacts of agricultural production systems, agricultural input efficiency, and food choice. Environ. Res. Lett. 2017; 12(6): 064016. doi: 10.1088/1748-9326/aa6cd5
5. Global Livestock Environmental Assessment Model (GLEAM) [Internet]. Food and Agriculture Organization. 2017. Available from: www.fao.org/gleam/en/
6. Poore J and Nemecek T. Reducing food's environmental impacts through producers and consumers. Science. 2018;360: 987–92. doi: 10.1126/science.aaq0216

7. Nijdam D, Rood T and Westhoek H. The price of protein: Review of land use and carbon footprints from life cycle assessments of animal food products and their substitutes. Food Policy. 2012;37: 760–70. doi: 10.1016/j.foodpol.2012.08.002
8. Capper J L and Bauman D E. The Role of Productivity in Improving the Environmental Sustainability of Ruminant Production Systems. Annu. Rev. Anim. Biosci. 2013;1: 469–89. doi: 10.1146/annurev-animal-031412-103727
9. Godde C M, de Boer I J M, Ermgassen E zu, Herrero M, van Middelaar C E, Muller A, et al. Soil carbon sequestration in grazing systems: managing expectations. Clim. Change. 2020;161: 385–91. doi: 10.1007/s10584-020-02673-x
10. Wang G, Zhang W, Sun W, Li T and Han P. Modeling soil organic carbon dynamics and their driving factors in the main global cereal cropping systems. Atmos. Chem. Phys. 2017;17: 11849–59. doi: 10.5194/acp-17-11849-2017
11. Stanley P L, Rowntree J E, Beede D K, DeLonge M S and Hamm M W. Impacts of soil carbon sequestration on life cycle greenhouse gas emissions in Midwestern USA beef finishing systems. Agric. Syst. 2018:162 249–58. doi: 10.1016/j.agsy.2018.02.003
12. McSherry M E and Ritchie M E. Effects of grazing on grassland soil carbon: a global review. Glob. Chang. Biol. 2013;19: 1347–57. doi: 10.1111/gcb.12144
13. Hayek M N, Harwatt H, Ripple W J and Mueller N D. The carbon opportunity cost of animal-sourced food production on land. Nat. Sustain. 2020;10–3. doi: 10.1038/s41893-020-00603-4
14. Searchinger T D, Wirsenius S, Beringer T and Dumas P. Assessing the efficiency of changes in land use for mitigating climate change. Nature. 2018;564: 249–53. doi: 10.1038/s41586-018-0757-z

15. Balmford A, Amano T, Bartlett H, Chadwick D, Collins A, Edwards D, et al. The environmental costs and benefits of high-yield farming. Nat. Sustain. 2018; 1: 477–85. doi: 10.1038/s41893-018-0138-5

16. Schmidinger K and Stehfest E. Including CO2 implications of land occupation in LCAs—method and example for livestock products. Int. J. Life Cycle Assess. 2012;17: 962–72. doi: 10.1007/s11367-012-0434-7

17. Pelletier N, Pirog R, Rasmussen R. Comparative life cycle environmental impacts of three beef production strategies in the Upper Midwestern United States. Agric. Syst. 2010 Jul 1;103(6):380-9. doi: 10.1016/j.agsy.2010.03.009

18. Conant R T, Cerri C E P P, Osborne B B and Paustian K. Grassland management impacts on soil carbon stocks: A new synthesis. A Ecol. Appl. 2017; 27: 662–8. doi: 10.1002/eap.1473

19. Garnett T, Godde C, Muller A, Röös E, Smith P, De Boer I, et al. Grazed and confused? Ruminating on Cattle, Grazing Systems, Methane, Nitrous Oxide, the Soil Carbon Sequestration Question-and what it All Means for Greenhouse Gas Emissions. Food Climate Research Network. 2017. Available from: https://www.fcrn.org.uk/sites/default/files/project-files/fcrn_gnc_report.pdf

20. Smith P, Bustamante M, Ahammad H, Clark H, Dong H, Elsiddig E A, et al. Agriculture, Forestry and Other Land Use (AFOLU). Climate Change 2014: Mitigation of Climate Change. Contribution of Working Group III to the Fifth Assessment Report of the Intergovernmental Panel on Climate Change. [R Edenhofer, O., J Pichs-Madruga, Y. Sokona, E. Farahani, S. Kadner, K. Seyboth, A. Adler, I. Baum, S. Brunner, P. Eickemeier, B. Kriemann and T Z and J C M Savolainen, S. Schlömer, C. von Stechow

(eds.)] (Cambridge, United Kingdom and New York, NY, USA: Cambridge University Press). 2014 Available at: http://www.ipcc.ch/pdf/assessment-report/ar5/wg3/ipcc_wg3_ar5_chapter11.pdf

21. West P C, Gibbs H K, Monfreda C, Wagner J, Barford C C, Carpenter S R et al. Trading carbon for food: global comparison of carbon stocks vs. crop yields on agricultural land Proc. Natl. Acad. Sci. U. S. A. 2010; 107: 19645–8. doi: 10.1073/pnas.1011078107
22. Veldman JW, Overbeck GE, Negreiros D, Mahy G, Le Stradic S, Fernandes GW, Durigan G, Buisson E, Putz FE, Bond WJ. Where tree planting and forest expansion are bad for biodiversity and ecosystem services. BioScience. 2015 Oct 1;65(10):1011-8. doi: 10.1093/biosci/biv118
23. Clark T S and Linzer D A. Should I Use Fixed or Random Effects? Polit. Sci. Res. Methods. 2015;3: 399–408. doi: 10.1017/psrm.2014.32
24. Hertel T W, Ramankutty N and Baldos U L C. Global market integration increases likelihood that a future African Green Revolution could increase crop land use and CO2 emissions. Proc. Natl. Acad. Sci. U. S. A. 2014;111: 13799–804. doi: 10.1073/pnas.1403543111
25. Blaustein-Rejto D, Blomqvist L, McNamara J and De Kirby K. Achieving Peak Pasture: Shrinking Pasture’s Footprint by Spreading the Livestock Revolution. The Breakthrough Institute. 2019.

Supporting information

S1 File: Supplementary methods, figures and tables.

Figure 1

Click here to access/download;Figure;Fig_1.tiff

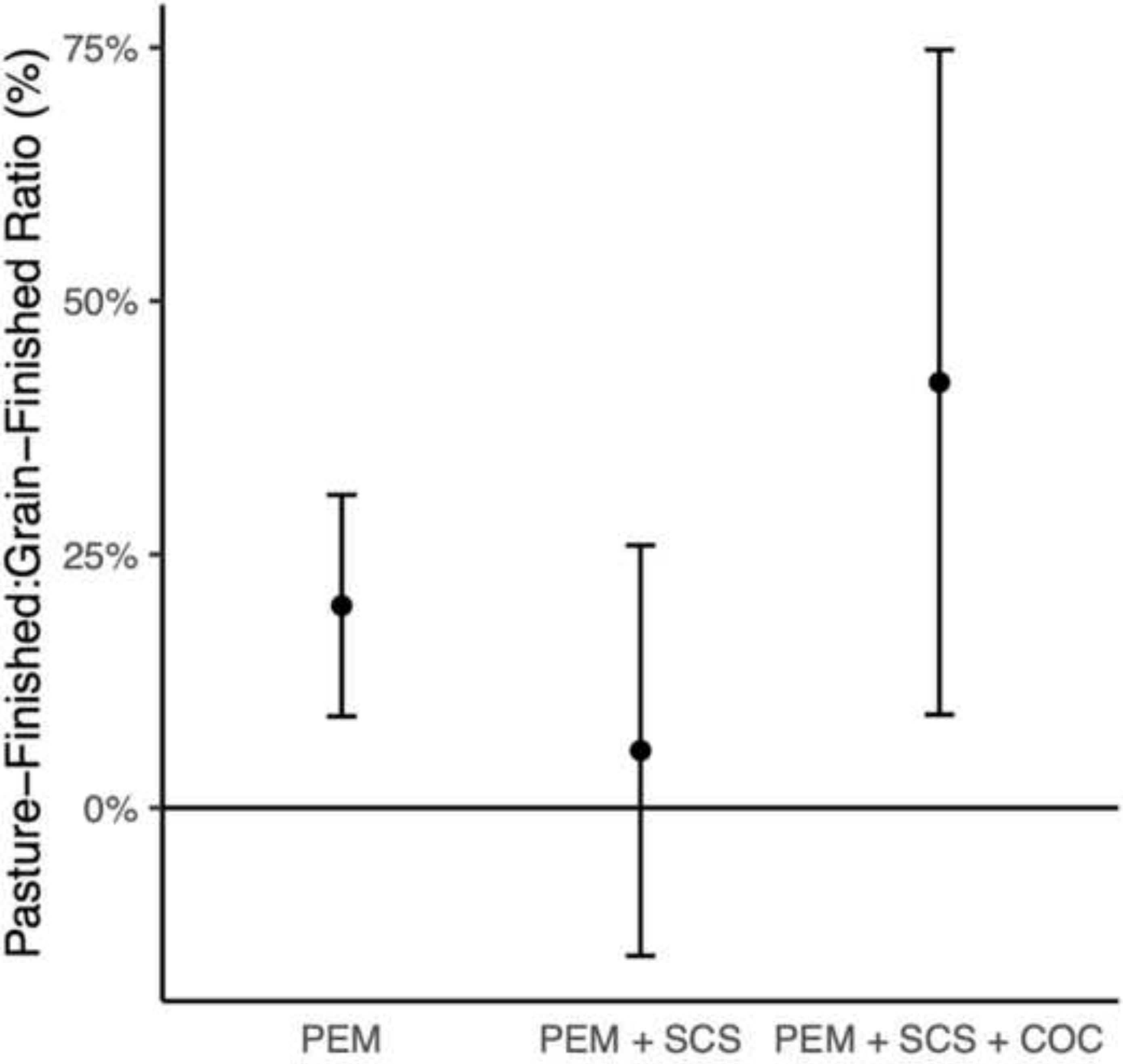

Revised Figure 2

Click here to access/download;Figure;Fig_2.tiff

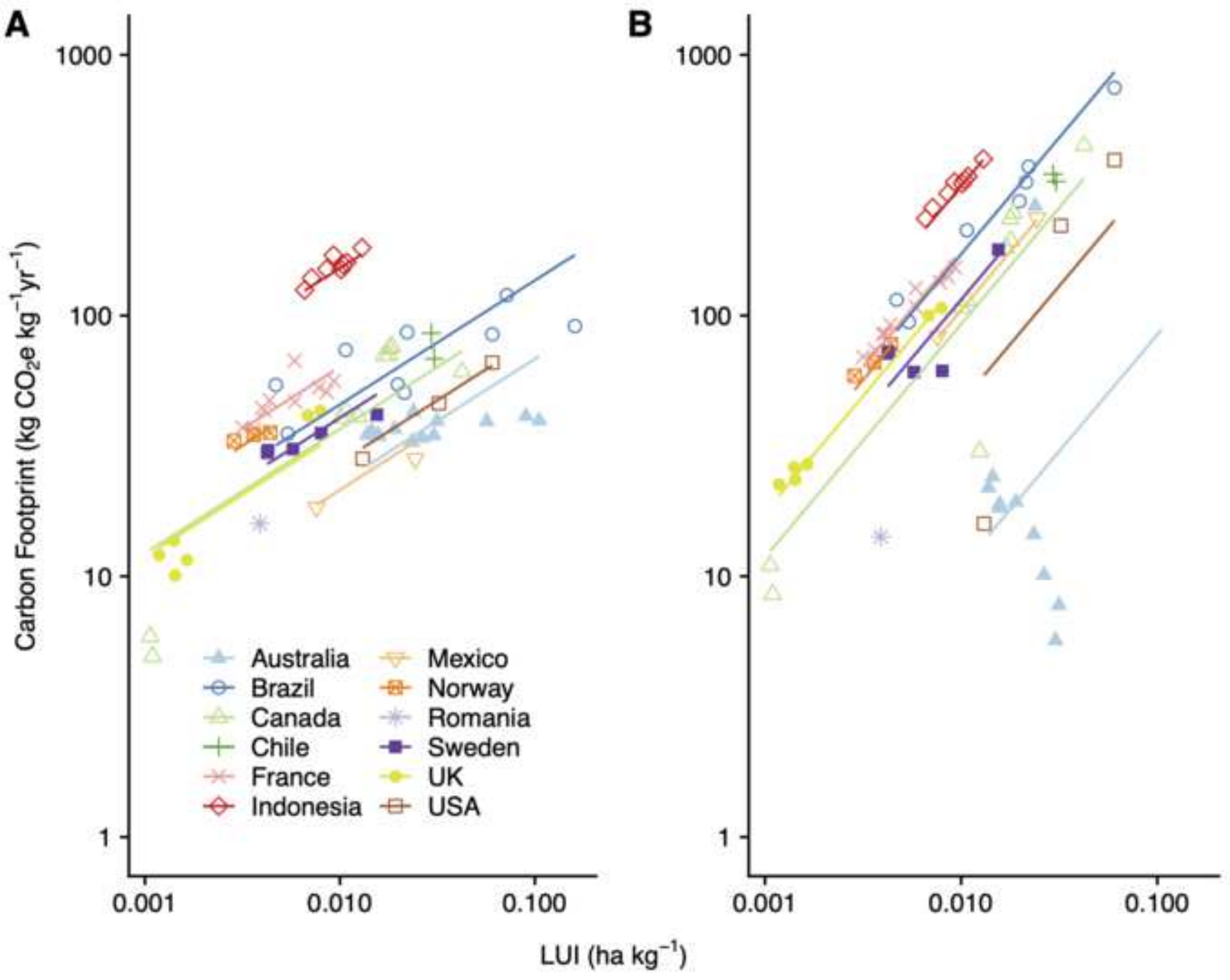

Click here to access/download
Supporting Information
supplementary_info_plos_one_clean.docx

Click here to access/download
Supporting Information
supplementary_info_plos_one_markup.docx

~~**Full Title:**~~ Carbon opportunity cost increases carbon footprint advantage of grain-finished beef

~~**Short Title:** Carbon footprint of grain- and grass-finished beef~~

Dan Blaustein-Rejto[1*], Linus Blomqvist[1,2], Nicole Soltis[3]

[1] The Breakthrough Institute, Berkeley, California, United States of America

[2] Bren School of Environmental Science & Management, University of California, Santa Barbara, California, United States of America

[3] California Council on Science and Technology, Sacramento, California, United States of America

*Corresponding author

~~email~~Email: dan@thebreakthrough.org (DBR)

Abstract

Beef production accounts for the largest share of global livestock greenhouse gas emissions and is an important target for climate mitigation efforts. Most life-cycle assessments comparing the carbon footprint of beef production systems have been limited to production emissions. None also consider potential carbon sequestration due to grazing and alternate uses of land used for production. We assess the total carbon footprint of 100 beef production systems in 16 countries, including production emissions, soil carbon sequestration from grazing, and carbon opportunity cost—the potential carbon sequestration that could occur on land if it were not used for production. We conduct a pairwise comparison of pasture-finished operations in which cattle almost exclusively consume grasses and forage, and grain-finished operations in which cattle are first grazed and then fed a grain-based diet. We find that pasture-finished operations have 20% higher production emissions and 42% higher total carbon footprint than grain-finished systems. We also find that more land-intensive operations generally have higher carbon footprints. Regression analysis indicates that a 10% increase in land-use intensity is associated with a 4.8% increase in production emissions, but a 9.0% increase in the total carbon footprint, including production emissions, soil carbon sequestration and carbon opportunity cost. The carbon opportunity cost of operations was, on average, 130% larger than production emissions. These results point to the importance of accounting for carbon opportunity cost in assessing the sustainability of beef production systems and developing climate mitigation strategies.

Introduction

Beef production accounts for about 6% of all anthropogenic greenhouse gas emissions [1]. Given rising demand in developing countries, reducing the greenhouse-gas (or carbon) footprint

of production, measured as kilograms carbon dioxide-equivalent (CO_2e) per kilogram~~kg~~ of beef, is an important climate mitigation strategy [2-3].

Whether beef is produced in pasture-finished or grain-finished systems affects its carbon footprint. In both pasture-finished and grain-finished systems, cattle are raised initially on pasture or rangeland. The primary difference lies in the finishing stage—in grain-finished systems, cattle are fed a grain-based diet and often kept in feedlots, whereas cattle in pasture-finished systems continue to eat fresh and stored grasses and hay until they reach slaughter weight [4]. The finishing stage therefore accounts for any potential difference in the carbon footprint of these systems. Pasture-finished systems are common in many parts of the world and account for approximately 33% of global beef production. Grain-finished systems account for 15%, and other systems, such as mixed crop-livestock production, account for the remainder [~~4~~5].

Most life-cycle assessments ~~comparing~~ of the carbon footprint of grain-finished and pasture-finished systems have been limited to emissions directly attributable to cradle-to-farmgate activities (here referred to as production emissions) [6]. Reviews and meta-analyses of these studies conclude that pasture-finished systems have a higher average carbon footprint [4,6,7~~5-7~~]. Grain finishing typically leads to much higher growth rates. As a result, proportionally less energy is expended on maintenance rather than growth, such that inputs and emissions per unit of beef is lower [8].

In addition to emissions associated with production, beef's carbon footprint is also influenced by land use. Recent meta-analyses show that pasture-finished systems have higher land-use intensity (measured as area per unit production) on average, since the amount of pasture

needed in the finishing stage of pasture-finished cattle is much larger than the amount of cropland needed to provide grain for the finishing stage of grain-finished cattle [46~~-7~~].

Greater land requirements influence the carbon footprint in two ways. First, pasture and crop management can increase soil carbon sequestration [9-10]. Use of improved grazing practices in some pasture-finished systems has sequestered enough carbon to offset production emissions from finishing [11]. Yet large soil carbon sequestration rates are only possible under particular agro-ecological conditions and for a limited time period [9,12].

Second, greater land use for beef production can displace native ecosystems and reduce land available for restoration. The amount of CO_2 that could be removed on land used for production through reforestation or other restoration has been referred to as the "carbon opportunity cost" [13].

Existing global comparisons of pasture-finished and grain-finished systems are incomplete as they do not account for both carbon opportunity cost and soil carbon sequestration. For instance, Poore and Nemecek (2018) [6~~7~~], in a global meta-analysis of life-cycle assessments, do not account for potential soil carbon sequestration from production or the carbon opportunity cost of land use. The authors do account for emissions from land-use change, but only from recent changes in which total area for the crop or livestock product increased in the country of production. This approach, unlike the carbon opportunity cost approach, can result in zero carbon costs associated with many types of land use (see Searchinger ~~*et al*~~ *et al.* 2018 [14] Supplementary Discussion for a detailed treatment). Balmford ~~*et al*~~ *et al.* (2018) [15~~4~~] estimate the relationship between the carbon footprint and land-use intensity of beef production including foregone carbon sequestration from land use—finding that there is a strong positive correlation—but their analysis is limited to Latin America and does not estimate soil carbon

sequestration from grazing. Schmidinger and Stehfest (2012) [~~15~~16], Searchinger ~~*et al*~~ *et al.* (2018) [~~16~~14], and Hayek ~~*et al*~~ *et al.* (2020) [13] estimate the carbon opportunity cost of beef production at different geographic scales, but do not compare grain-finished and pasture-finished systems or estimate soil carbon sequestration from grazing.

Here, for the first time, we assess the total carbon footprint – defined as the sum of carbon emissions from production, soil carbon sequestration, and carbon opportunity cost – of pasture-finished and grain-finished systems from across the world. We compare the total carbon footprint of pasture-finished and grain-finished systems that exist in the same region and that have been studied using the same methodology.

~~We find that grain-finished production has a significantly smaller total carbon footprint than pasture-finished production. Pasture-finished systems have higher average production emissions than grain-finished systems. Incorporating soil carbon sequestration reduces the carbon footprint gap between pasture-finished and grain-finished systems. But when carbon opportunity cost is also included, pasture-finished systems have a 42% larger carbon footprint than grain-finished systems.~~

~~The lower land-use intensity of grain-finished systems is a large factor in their lower carbon footprint.~~ To ~~confirm that this~~assess the relationship between land-use intensity and carbon footprint ~~holds in general~~, regardless of the system, we also regress several carbon footprint measures on land-use intensity. ~~This shows that a 10% increase in land-use intensity is associated with a 4.8% increase in production emissions, and a 9% increase in total carbon footprint, including soil carbon sequestration and carbon opportunity cost.~~

Beef production systems are changing rapidly across the world, and decisions about the future direction of this change will have important implications for climate mitigation as well as

other environmental impacts. Accounting for the total carbon footprint, including the carbon opportunity cost, as we do in this paper, should help guide these decisions.

Materials and methods

We calculate the total carbon footprint (the sum of production emissions, soil carbon sequestration, and carbon opportunity costs in kilograms~~kg~~ CO_2e per kilogram~~kg~~ of retail weight beef) of 100 beef production operations across 16 countries, including those from beef and dairy herds, drawn from a dataset of food and beverage life-cycle assessments [9] and from Stanley ~~*et al*~~ *et al.* (2018) [11]. Poore and Nemecek (2018) [9] includes production emissions and land-use intensity data. Stanley ~~*et al*~~ *et al.* (2018) [11] reports production emissions, carbon sequestration, emissions from soil erosion, and land-use intensity for the finishing stage of a pasture-finished and grain-finished operation in the Midwestern USA; we derive values from earlier stages from Pelletier ~~*et al*~~ *et al.* (2010) [17] which also studied operations in the Midwest. We conduct a pair-wise comparison of carbon footprints between pasture-finished and grain-finished beef production systems, and a regression analysis of the relationship between land-use intensity and carbon footprint.

Production emissions ~~&~~ and land-use intensity

Production emissions represent cradle-to-farmgate life-cycle greenhouse gas emissions. This includes emissions associated with enteric fermentation, animal housing, manure management, and inputs associated with feed production such as fertilizers, pesticides, and machinery.

Land-use intensity represents land required for grazing and crop production, in hectare~~ (ha)~~ per kilogram~~kg~~ of retail weight beef. Land use for pasture is calculated as the sum of temporary

and permanent pasture, and land use for cropland is calculated as the sum of seed, arable and fallowed crop land. We use and standardize production emissions and land-use intensity values from Poore and Nemecek (2018) [9] and Stanley ~~*et al*~~ *et al.* (2018) [11].

Soil carbon sequestration

Soil carbon sequestration (SCS) in kg CO_2 per kg of retail weight beef is calculated as the product of land-use intensity of grazing (LUI) and carbon sequestration due to grazing (CSG) in kg C ha^{-1} yr^{-1} (Equation 1).

$$SCS = LUI \cdot CS \cdot \frac{44\ CO_2}{12\ C} \qquad (1)$$

There is insufficient data to calculate a specific carbon sequestration rate for each life-cycle assessment location. This is in part because sequestration rates depend on environmental and management factors, such as soil texture and grazing intensity, not consistently described in the life-cycle assessments. Instead, for all life-cycle assessments we use the mean carbon sequestration rate of 0.28 Mg ~~carbon (C)~~C ha^{-1} yr^{-1} for “improved grazing management” estimated in a synthesis of the grassland management literature [18]. This estimate, drawn from studies with an average soil depth of 23 cm, is within the range of peer reviewed estimates: 0.03 and 1.04 Mg C $ha^{-1}yr^{-1}$, with the lowest values corresponding to dry climates and the highest to specific grassland management practices and regions [19]. Given that not all the life-cycle assessments included are of operations with improved grazing practices, the true carbon sequestration rates across operations may be lower. To be conservative in our carbon footprint for grain-finished operations, we assume that no carbon sequestration occurs on cropland used for feed production, consistent with research that shows that CO_2 emissions from agricultural land are generally balanced by removals [20].

Carbon opportunity cost

Our measure of carbon opportunity cost calculates how much carbon sequestration would have occurred had land been occupied with native ecosystems instead of pasture or cropland. This assumes that reducing land-use intensity results in proportionately less agricultural land area locally.

We calculate carbon opportunity cost (COC) as the sum of the carbon opportunity cost of pasture (*p*) and cropland (*c*) used in production. For each of these two land uses, the carbon opportunity cost is calculated as the product of land-use intensity (LUI) and potential carbon sequestration (PCS) of the land in the area where the life-cycle assessments was conducted, in kg C ha^{-1} yr^{-1} (Equations 2 and 3).

$$COC = \sum_i LUI_i \cdot PCS_i \cdot \frac{44\ CO_2}{12\ C} \text{ for } i = c, p \qquad (2)$$

Wherewhere

$$PCS_i = \frac{NPP_i \cdot k_i \cdot r - s_i}{r} \text{ for } i = c, p \qquad (3)$$

NPP_i denotes the potential net primary productivity of native vegetation (kg C ha^{-1} yr^{-1}) that could be restored on agricultural land within a given radius of where the life-cycle assessment was conducted. We report results using a radius of 2 degrees (~223 km at equator). k_i is the conversion factor from net primary productivity to carbon sequestration in vegetation and soils or, put differently, the average level of carbon sequestration generated by devoting one kilogramkg of NPP to restoring native vegetation. This value is 0.42 kg CO_2 ha^{-1} yr^{-1} for every kg of NPP for cropland and 0.44 for pasture, as calculated by Searchinger et al et al. (2018) [14]. r denotes the time period over which carbon sequestration is averaged, in this case 100 years; and s_i denotes existing vegetation carbon stocks (kg C ha^{-1}), 1100 for cropland and 3100 for pasture, based on global averages for cereals and pasture, respectively, from Searchinger et al et al.

(2018) [14]. Although spatially explicit estimates of cropland carbon stocks exist [21], we are not aware of any for pasture carbon stocks.

The logic behind Equation 3 is as follows. The numerator represents the difference in potential carbon stocks between current land use and native vegetation. $NPP_i \cdot k_i$ is a flux measure, in kilograms~~kg~~ of carbon per hectare per year, which we multiply by 100 to turn into a stock measure. In effect, this assumes that the equilibrium carbon stock in native ecosystem is reached after 100 years. The numerator, the difference in potential carbon stocks, is then divided by 100 to arrive at an annual (flux) rate. We select a time period of 100 years based on previous studies such as Searchinger ~~*et al*~~ *et al.* (2018) [14] and Schmidinger and Stehfest (2012) [16], which use it as a time period over which to calculate average carbon sequestration rates in regenerating forests.

Data on potential net primary productivity under native vegetation is generated by the Lund–Potsdam–Jena managed Land (LPJmL) model, a dynamic global vegetation model that simulates vegetation composition, distribution, and carbon stocks and flows at 0.5x0.5° spatial resolution. We use LPJmL results from Searchinger ~~*et al*~~ *et al.* (2018) [14].

We assume life-cycle assessment sites located in climate categorized as “dry” in Poore & Nemecek (2018) [9] have zero potential carbon sequestration because they either cannot support substantial additional biomass or are native grasslands or savannas for which restoration does not typically involve reforestation [22].

Pairwise comparison between pasture-finished and grain-finished production systems

We compare the carbon footprint of 20 pairs of pasture-finished and grain-finished production systems, across 12 countries, in the Poore and Nemecek (2018) [9] database and one recent comparative life-cycle assessment [11] with and without soil carbon sequestration and carbon opportunity cost included. Systems were selected for inclusion if they were in the same subnational region or country, if the study was national in scope, and reported in the same study or within two studies by the same primary author. Details of the operations pairs are listed in S8 Table S8. Fourteen of the pairs were reported for the same geographic region, but lacked coordinates. For those, we estimated carbon opportunity cost by calculating mean potential net primary productivity on cropland and grazing land within the subnational region or country the life-cycle assessment was located (Supplementary Methods). We used a paired t-test to test if the mean difference between the pasture-finished and grain-finished system was significantly different from zero.

Regression analysis

We also assess the relationship between carbon footprint and land-use intensity using cross-section regression analysis of beef production operations. We include 72 operations from life-cycle assessments that report geographic coordinates, including a total of 24 studies in 12 countriesWe only include life-cycle assessments with coordinates for the location of the analyzed beef production system, a total of 72 operations across 24 studies in 12 countries (S1 Fig S1, S7 Table S7). We log-transform the carbon footprint and land-use intensity because the input data is heavily right-skewed and because this enables us to present results as elasticities—the expected

percent change in the carbon footprint with a percentage change in land-use intensity. This yields the following regression equation:

$$log(carbon\ footprint) = +log(LUI) + \epsilon \qquad (4)$$

Where the parameter of interest, , represents the elasticity between land-use intensity and the carbon footprint, and ϵ is an error term.

We run three different regressions, starting with production emissions as the only regressor, adding carbon opportunity cost in the second regression, and then also including soil carbon sequestration in the third regression. We use a linear model to facilitate comparison of the relationship across the regressions. Since there may be variables operating at the country level that influence the carbon footprint (e.g. climate, national policy), we use a multilevel model with country-level random effects, particularly varying intercepts and constant slopes (Gelman and Hill, 2007). we consider a fixed effects or random effects model. This yields the following regression equation:

$$log(carbon\ footprint_{i,j}) = \beta_0 + \beta_1 log(LUI_{i,j}) + u_j + \epsilon_{i,j} \qquad (4)$$

where j indexes countries, i indexes operations within countries, $\beta_0 + u_j$ is the intercept for each country, β_1 represents the elasticity between land-use intensity and the carbon footprint, and ϵ_{ij} is an error term.

We choose this specification over a fixed effect model as there is substantial variation in the independent variable within units (i.e. countries), the level of correlation between unit effects and the independent variable is not extremely high, and we are interested in accounting for the variability between units but not in estimating specific unit effects, in which case a random effects model can be appropriate to use and result in superior estimates (Clark and Linzer 2015). Random-effects estimators require that there be no correlation between the covariate of interest

Formatted: Indent: Left: 0", First line: 0.3"

Formatted: Indent: Left: 0", First line: 0.3"

Formatted: Font: Italic, Subscript

~~(here, $log(LUI)$) and the unit effects (in this case, $country$). A Hausman test on the specification with production emissions and carbon opportunity cost included suggests that the correlation is relatively low, motivating our choice of a random-effects model. This is further justified by the relatively small sample size, in which case a random-effects model is likely superior to a fixed-effects model [23].~~ Regressions with fixed effects produced results very similar to those with random effects (S5 Table S5). Our analysis examines differences in ~~net~~ total carbon footprint across operations with different land-use intensity and does not attempt causal inference per se.

Robustness checks

We vary four parameters to assess the robustness of the results. First, we run the analysis with 0.25, 0.5, 1.0 and 4 degree radius. We do this to confirm our results cannot be explained by the choice of radius as NPP values can vary widely over a small area.

Second, we run the analysis with alternative calculations for carbon opportunity cost at the national and global levels. The national and global carbon opportunity costs assume that if the amount of land needed to support a given level of food production declines by one unit as a result of lower land-use intensity, then one unit of land will be restored to native vegetation somewhere in the country or world, respectively. These are relevant comparisons in cases where domestic and international trade allow land-use intensity reductions to be spatially disconnected from pasture and cropland expansion/contraction. We calculate national carbon opportunity cost using the average NPP values over all crop and pasture land across the country each production system is located in. This method could be improved by using crop-specific values; however, not all life-cycle assessments in our dataset describe which crops are used in production. We also calculate global carbon opportunity cost using average global net primary production values.

Third, we run the analysis using a carbon sequestration rate of 0.47 Mg C ha^{-1} yr^{-1}, the average value reported across all studies of improved grassland management included in Conant ~~*et al*~~ *et al.* (2017). This reduces the total carbon footprint of more land-intensive operations such as pasture-finished systems more than it reduces the carbon footprint of less land-intensive operations.

Fourth, we run the analysis with and without the potential carbon sequestration, and thus the carbon opportunity cost, set to 0 for operations in dry climates.

Results

In this study we calculated the total carbon footprint of beef production systems as the sum of production emissions, carbon opportunity cost, and soil carbon sequestration, and assessed the relationship of total carbon footprint and land-use intensity. After presenting summary statistics, we show the results of the pair-wise comparison of the carbon footprints of pasture-finished and grain-finished beef production systems. We then present results from regression analysis of carbon footprints on land-use intensity, with separate regressions for the different approaches for calculating carbon footprint.

The total carbon footprint across the 72 beef production operations with reported latitude and longitude, and the 28 operations without latitude/longitude included in the pasture-finished/grain-finished comparison ranged from -68.3 to 2169.3 kg CO_2e kg^{-1} ~~/kg~~ retail weight, with mean 177.37 and median 107.14 (Table 1). Four pasture-finished and one grain-finished production systems in Queensland, Australia are estimated to have negative carbon footprints, in part because we assume that the dry climate results in zero carbon opportunity cost. If soil carbon sequestration rates are lower in dry climates than other climates, as some studies such as

Smith ~~et al~~ *et al.* (2008) suggest, these operations would be more likely to also have positive carbon footprints. The total carbon footprint was similar in robustness checks, with the mean value~~net carbon footprint~~ ranging from 141.6 to 210.0 kg CO_2e kg^{-1}~~/kg~~ retail weight when different radii are used and when we do not assume zero carbon opportunity cost for arid climates (S1 Table ~~S1~~).

Table 1: Summary statistics for beef operations~~.~~

Variable	Mean	Median	Range	95% CI	Units
Production emissions	52.64	41.42	4.9, 182	45.48, 59.8	kg CO_2e kg^{-1}
Soil carbon sequestration	-15.11	-7.41	-164.8, 0	-19.96, -10.26	kg CO_2e kg^{-1}
Carbon opportunity cost	139.85	68.46	0, 2243	87.1, 192.59	kg CO_2e kg^{-1}
Total carbon footprint	177.37	107.14	-68.3, 2169.3	124.79, 229.96	kg CO_2e kg^{-1}
Land-use intensity	0.02	0.01	0, 0.2	0.01, 0.02	ha kg^{-1}

All units are per kilogram retail weight. n = 100.

In individual systems, carbon opportunity cost was, on average, 130% larger than production emissions. Soil carbon sequestration offset 31.5% of production emissions and 18.9% of the production emissions and carbon opportunity cost, on average. Across all robustness checks, carbon opportunity cost is at least 65% larger than production emissions and soil carbon sequestration does not fully offset production emissions (S2 Table ~~S2~~).

Formatted: Superscript

Formatted Table

Pairwise comparison between pasture-finished and grain-finished systems

The pairwise comparison found that pasture-finished systems had 20% higher mean production emissions than grain-finished systems on average ($p<0.01$). When also including soil carbon sequestration, the difference is not statistically significant at a 95% confidence level ($p\geq0.05$). When the carbon opportunity cost is also accounted for, however, the total carbon footprint of pasture-finished systems is on average 42% higher than that of grain-finished systems ($p<0.01$) (Fig 1~~, Table S3~~). Compared to grain-finished systems, pasture-finished systems also had 15% higher median production emissions ($p<0.01$) and total carbon footprints ($p<0.05$), indicating that while the magnitude of the difference is sensitive to extreme values, the general finding of higher emissions is robust (S3 Table~~ S3~~).

Fig 1: Average ratios of carbon footprints between pasture-finished and grain-finished. Ratios expressed as percentage difference. PEM denotes production emissions, SCS denotes soil carbon sequestration, and COC denotes carbon opportunity cost. Values above (below) 0 denote the carbon footprint for pasture-finished operations is larger (smaller) than for grain-finished operations. Comparisons were made within paired production systems to control for agronomic and environmental differences. Bars show means and 95% confidence intervals. On average, carbon footprints for pasture-finished operations are significantly greater ($p<0.01$) than those of grain-finished operations when only production emissions are included and when production emissions, soil carbon sequestration and carbon opportunity cost are included. n = 20 pairs.

The carbon footprint of pasture-finished systems, including production emissions, soil carbon sequestration and carbon opportunity cost, is higher than that of the grain-finished systems ($p<0.05$) in the majority of robustness tests (S4 Table ~~S4~~). Differences are not significant ($p\geq0.05$) in some cases when a smaller radius or higher rate of soil carbon sequestration is used.

Regression analysis

In the regression analysis, when only production emissions are regressed on land-use intensity, the coefficient is 0.48 (Fig 2a, Table 2). This can be interpreted as a 10% increase in land-use intensity being associated with a 4.8% increase in carbon footprint. Less land-intensive systems typically have lower carbon footprints, measured by production emissions alone. Fig 2a shows the regression line with this slope, with the level adjusted by country. When adding in soil carbon sequestration, the coefficient is reduced to 0.32, indicating that soil carbon sequestration offsets a part of the production emissions (Table 2).

~~However, the relationship between total carbon footprint, including carbon opportunity cost, and land-use intensity is stronger, with a coefficient of 0.90 (Table 2, Fig 2b). Hence, a 10% increase in land-use intensity is associated with a 9.0% increase in the total carbon footprint of beef production. This near-proportional relationship is in part due to the large share of the total carbon footprint accounted for by carbon opportunity cost, which is proportional to land area in production.~~

~~Regressions with pooled and country fixed-effects specifications generate similar results (Table S5). Results are robust to other specifications and assumptions checked (Table S6).~~

Fig 2: The relationship between land-use intensity and carbon footprint of beef production systems. Results from a regression of log(carbon footprint) on log(land-use intensity) with country random effects. Dots indicate life-cycle assessment observations; colors indicate countries; and lines represent the slope of the regression that includes all countries, adjusted according to the levels of each country. A) Carbon footprint including only production emissions. *n* = 72. B) Carbon footprint including production emissions, soil carbon sequestration and carbon opportunity cost. *n* = 69.

Table 2: Results from log-log regressions

	Dependent variable:		
	PEM	PEM+SCS	PEM+SCS+COC
LUI	0.48***	0.32***	0.90***
	(0.04)	(0.08)	(0.09)
Constant	5.90***	4.84***	8.70***
	(0.27)	(0.45)	(0.52)
Observations	72	68	69
R^2	0.67	0.27	0.63
Adjusted R^2	0.66	0.25	0.63

~~*Note:*~~
Standard errors in parentheses. LUI = land-use intensity. PEM = production emissions. SCS = soil carbon sequestration. COC = carbon opportunity cost.
* p < 0.01,; ** p < 0.05,; *** p < 0.01

However, the relationship between total carbon footprint, including carbon opportunity cost, and land-use intensity is stronger, with a coefficient of 0.90 (Table 2, Fig 2b). Hence, a 10% increase in land-use intensity is associated with a 9.0% increase in the total carbon

Formatted: Left, Border: Top: (No border), Bottom: (No border), Left: (No border), Right: (No border), Between : (No border)

Commented [A1]: The extra 0 was a typo introduced by the authors when converting the image of the table, generated programmatically in R, to text in Word prior to submission. This is now corrected. We regret the error and note that it was limited to this line of the table only, and does not affect or apply to any of the text or results presented.

footprint of beef production. This near-proportional relationship is in part due to the large share of the total carbon footprint accounted for by carbon opportunity cost, which is proportional to land area in production.

Regressions with pooled and country fixed-effects specifications generate similar results (S5 Table S5). Results are robust to other specifications and assumptions checked (S6 Table S6).

Formatted: Left

Discussion

Our analysis is the first global comparison of the carbon footprint of grain-finished and pasture-finished beef production systems that includes production emissions as well as soil carbon sequestration and carbon opportunity cost. This yields significant new insights that can inform environmental and agricultural decision-making.

Our results indicate that pasture-finished and other more land-intensive beef production systems have greater production emissions than grain-finished and less land-intensive systems. When we calculate carbon footprints including production emissions, soil carbon sequestration, and carbon opportunity cost, all beef production systems have a higher carbon footprint than when only production emissions are included, but pasture-finished systems have a substantially larger carbon footprint than grain-finished systems, and there is a strong positive relationship between land use intensity and carbon footprint.

The differences in carbon footprint between pasture- and grain-finished operations are largely due to differences in carbon opportunity cost, which account for a large share of the total carbon footprint. The carbon opportunity cost of operations was, on average, 130% larger than

production emissions. These results point to the importance of accounting for carbon opportunity cost in assessing the sustainability of beef production systems.

Our analysis also confirms that beef operations that have been studied in life-cycle assessments are generally not carbon neutral or negative. The mean carbon footprint across all studies, including production emissions, sequestration, and carbon opportunity cost, is over three times larger than the mean value for production emissions (Table 1). One exception is that we estimate negative carbon footprints for several grass-finished operations and one grain-finished operation that are in dry eco-climate zones in Australia, for which we assume there is zero carbon opportunity cost. This suggests that grazing cattle on dry rangeland with little to no carbon opportunity cost could have a small carbon footprint when the grazing also increases soil organic carbon, as has been observed in some studies of dry rangeland with finer textured soil [12].

Our comparison of pasture-finished and grain-finished systems builds upon and strengthens past findings. Our finding that production emissions are 20% higher on pasture-finished operations than on grain-finished operations is consistent with Clark and Tilman (2017) [6], which found average emissions were 19% higher though their estimate was not statistically significant. In our results, soil carbon sequestration from grazing offsets only a portion of production emissions. This finding is consistent with the conclusions of Garnett ~~et al~~ *et al.* (2017) [19], which estimated that soil carbon sequestration from grazing can offset 20-60% of annual emissions from ruminant grazing.

Our finding that land-use intensity and carbon footprint are positively correlated strengthens similar findings from previous studies, none of which included production emissions, soil carbon sequestration and carbon opportunity cost, which is a more comprehensive approach for

assessing the carbon footprint of land use than conventional land-use change approaches [16]. Poore and Nemecek (2018) [9] found that beef and lamb systems with lower land-use intensity have a lower carbon footprint when considering land-use change-related greenhouse-gas emissions, but not carbon opportunity cost. Balmford ~~*et al*~~ *et al.* (2018) [14] used generalized linear mixed models to analyze the relationship between land-use intensity and carbon footprint, including a measure of carbon opportunity cost based on IPCC (2006) methods. Their analysis, limited to Brazil and tropical Mexico, also found that the carbon opportunity cost of agriculture was typically greater than production emissions, and that incorporating opportunity costs generated strongly positive associations between carbon footprint and land-use intensity. Searchinger ~~*et al*~~ *et al.* (2018) [14] calculated global-average carbon opportunity costs for beef similar to the average calculated for all operations included in this study. Their estimates of 165.3 and 143.9 kg CO_2e kg^{-1} carcass weight were based on the potential carbon that could be gained or lost, respectively, on land used for production. The authors applied the values to five production systems in Brazil and found, consistent with our results, that systems with the lowest land-use intensity had the greatest carbon benefits.

Our study has several limitations although we do not believe these substantially alter our conclusions. The pairwise comparison of grain-finished and pasture-finished operations has a relatively small sample of 20 pairs. This means that assumptions of asymptotic normality, which are the basis for the paired t-test, may not hold. However, our robustness checks (S4 Table ~~S4~~) and nonparametric test of the median (S3 Table ~~S3~~), which is robust to small sample sizes, extreme outliers, and heavy-tailed distributions, reinforce the conclusion that pasture-finished operations have greater production emissions and total carbon footprints than grain-finished operations. In addition, our results cannot be considered to be globally representative or

representative of all operations. The life-cycle assessments that underlie our study were not conducted to be globally representative. For instance, we include one study from Asia (Indonesia) and none from Africa.

In our study, we assume that a change in land-use intensity results in a proportionate change in land under production and thus the land area with native ecosystems. While this has the advantage of simplicity, it is unlikely to be exactly true in reality, as a result of economic mechanisms. The real effect may be more or less than proportional depending, in part, on how differences in land-use intensity and carbon footprint are associated with total factor productivity. For instance, an operation shifting from grain-finished to pasture-finished may lower total factor productivity. This would increase prices and lead to a reduction in overall demand, while at the same time making that operation less profitable and thus induce producers elsewhere to produce more. The reduction in demand would reduce land use and the spillover of production would increase land use, with an ambiguous net impact.

It is also challenging to predict where a change in farmland area and native vegetation will take place as a result of changes in land-use intensity and production system in a given location. We calculate three measures of carbon opportunity cost: local, national, and global. These roughly correspond to different levels of market connectedness, which will differ between locations. For example, changes in US production can have large effects on global markets, whereas changes in less globally connected regions such as sub-Saharan Africa will likely see mostly local or national effects [24]. Furthermore, for those producers connected to global markets, effects of changes in production are not likely to be evenly distributed across the world, but are likely to be concentrated in those regions that are more globally integrated [24]. In the last few decades, much of the expansion of pasture has taken place in tropical countries like

Brazil [25]. Following this logic, it is possible that higher land-use intensity in the US as a result of shifting to pasture-finished systems would displace production to these places, and is thus more likely to displace highly carbon-rich tropical ecosystems.

In addition, we use several simplifying assumptions. We use global mean estimates of soil carbon sequestration and current carbon stocks in cropland and grazing land vegetation due to lack of spatially-explicit data with global coverage. Our assumed rate is drawn from estimates for improved grazing management, so as to lessen the risk of overestimating the carbon footprint of grass-finished systems. Our measures of carbon opportunity cost are also based on mean potential carbon sequestration values in grazing land and cropland, if restored to native vegetation. They do not account for livestock diet rations, which crops are used for feed, or crop yields for instance. This may contribute to us underestimating potential carbon sequestration and carbon opportunity costs if feed crops such as soy are grown in areas with higher potential carbon sequestration, such as former forest, than other crops.

Future research could build upon our analysis by integrating more spatially explicit estimates of soil carbon sequestration and carbon stocks and calculating carbon opportunity cost based on how different cropland and grazing land is used in beef production. Further types of beef and other livestock operations, such as pork or milk, could also be studied with similar methods.

Overall, this study provides a novel assessment of the carbon footprint of beef operations, building upon life-cycle assessments of production emissions to also include carbon sequestration and carbon opportunity cost. Our conclusion that beef operations with low land-use intensity, including grain-finished operations, have lower total carbon footprints than pasture-finished operations and others with high land-use intensity provides important insights for

agricultural stakeholders. Accounting for products' total carbon footprint, not just production emissions, could shift which production systems government programs, corporate procurement, investors, and consumers incentivize.

Acknowledgements

The authors would like to thank Kenton de Kirby, Ken Cassman, and Joseph Poore for valuable comments on the draft manuscript.

References

1. Steinfeld H, Gerber P, Wassenaar T D, Castel V, Rosales M, Rosales M and de Haan C. Livestock’s long shadow: Environmental issues and options. 2006. Food and Agriculture Organization of the United Nations. Available at: https://www.fao.org/3/a0701e/a0701e.pdf
2. Clark M A, Domingo N G G, Colgan K, Thakrar S K, Tilman D, Lynch J, et al. Global food system emissions could preclude achieving the 1.5° and 2°C climate change targets. Science. 2020;370: 705–8. doi: 10.1126/science.aba7357
3. Swain M, Blomqvist L, McNamara J and Ripple W J. Reducing the environmental impact of global diets. Sci. Total Environ. 2018: 1207–9. doi: 10.1016/j.scitotenv.2017.08.125
4. Clark M and Tilman D. Comparative analysis of environmental impacts of agricultural production systems, agricultural input efficiency, and food choice. Environ. Res. Lett. 2017; 12(6): 064016. doi: 10.1088/1748-9326/aa6cd5

~~4.~~5. Global Livestock Environmental Assessment Model (GLEAM) [Internet]. Food and Agriculture Organization. 2017. Available from: www.fao.org/gleam/en/

6. Poore J and Nemecek T. Reducing food’s environmental impacts through producers and consumers. Science. 2018;360: 987–92. doi: 10.1126/science.aaq0216

~~5.~~7. Nijdam D, Rood T and Westhoek H. The price of protein: Review of land use and carbon footprints from life cycle assessments of animal food products and their substitutes. Food Policy. 2012;37: 760–70. doi: 10.1016/j.foodpol.2012.08.002

~~6.~~1. ~~Clark M and Tilman D. Comparative analysis of environmental impacts of agricultural production systems, agricultural input efficiency, and food choice. Environ. Res. Lett. 2017; 12(6): 064016. doi: 10.1088/1748-9326/aa6cd5~~

~~7.~~1. ~~Poore J and Nemecek T. Reducing food's environmental impacts through producers and consumers. Science. 2018;360: 987–92. doi: 10.1126/science.aaq0216~~

8. Capper J L and Bauman D E. The Role of Productivity in Improving the Environmental Sustainability of Ruminant Production Systems. Annu. Rev. Anim. Biosci. 2013;1: 469–89. doi: 10.1146/annurev-animal-031412-103727

9. Godde C M, de Boer I J M, Ermgassen E zu, Herrero M, van Middelaar C E, Muller A, et al. Soil carbon sequestration in grazing systems: managing expectations. Clim. Change. 2020;161: 385–91. doi: 10.1007/s10584-020-02673-x

10. Wang G, Zhang W, Sun W, Li T and Han P. Modeling soil organic carbon dynamics and their driving factors in the main global cereal cropping systems. Atmos. Chem. Phys. 2017;17: 11849–59. doi: 10.5194/acp-17-11849-2017

11. Stanley P L, Rowntree J E, Beede D K, DeLonge M S and Hamm M W. Impacts of soil carbon sequestration on life cycle greenhouse gas emissions in Midwestern USA beef finishing systems. Agric. Syst. 2018:162 249–58. doi: 10.1016/j.agsy.2018.02.003

12. McSherry M E and Ritchie M E. Effects of grazing on grassland soil carbon: a global review. Glob. Chang. Biol. 2013;19: 1347–57. doi: 10.1111/gcb.12144

13. Hayek M N, Harwatt H, Ripple W J and Mueller N D. The carbon opportunity cost of

animal-sourced food production on land. Nat. Sustain. 2020;10–3. doi: 10.1038/s41893-020-00603-4

14. Searchinger T D, Wirsenius S, Beringer T and Dumas P. Assessing the efficiency of changes in land use for mitigating climate change. Nature. 2018;564: 249–53. doi: 10.1038/s41586-018-0757-z

~~14.~~15. Balmford A, Amano T, Bartlett H, Chadwick D, Collins A, Edwards D, et al. The environmental costs and benefits of high-yield farming. Nat. Sustain. 2018; 1: 477–85. doi: 10.1038/s41893-018-0138-5

~~15.~~16. Schmidinger K and Stehfest E. Including CO2 implications of land occupation in LCAs—method and example for livestock products. Int. J. Life Cycle Assess. 2012;17: 962–72. doi: 10.1007/s11367-012-0434-7

~~16.~~1. ~~Searchinger T D, Wirsenius S, Beringer T and Dumas P. Assessing the efficiency of changes in land use for mitigating climate change. Nature. 2018;564: 249–53. doi: 10.1038/s41586-018-0757-z~~

17. Pelletier N, Pirog R, Rasmussen R. Comparative life cycle environmental impacts of three beef production strategies in the Upper Midwestern United States. ~~Agricultural~~ Agric. Syst~~ems~~. 2010 Jul 1;103(6):380-9. doi: 10.1016/j.agsy.2010.03.009

18. Conant R T, Cerri C E P P, Osborne B B and Paustian K. Grassland management impacts on soil carbon stocks: A new synthesis. A Ecol. Appl. 2017; 27: 662–8. doi: 10.1002/eap.1473

19. Garnett T, Godde C, Muller A, Röös E, Smith P, De Boer I, et al. Grazed and confused? Ruminating on Cattle, Grazing Systems, Methane, Nitrous Oxide, the Soil Carbon Sequestration Question-and what it All Means for Greenhouse Gas Emissions. Food

Climate Research Network. 2017. Available from: https://www.fcrn.org.uk/sites/default/files/project-files/fcrn_gnc_report.pdf

20. Smith P, Bustamante M, Ahammad H, Clark H, Dong H, Elsiddig E A, et al. Agriculture, Forestry and Other Land Use (AFOLU). Climate Change 2014: Mitigation of Climate Change. Contribution of Working Group III to the Fifth Assessment Report of the Intergovernmental Panel on Climate Change. [R Edenhofer, O., J Pichs-Madruga, Y. Sokona, E. Farahani, S. Kadner, K. Seyboth, A. Adler, I. Baum, S. Brunner, P. Eickemeier, B. Kriemann and T Z and J C M Savolainen, S. Schlömer, C. von Stechow (eds.)] (Cambridge, United Kingdom and New York, NY, USA: Cambridge University Press). 2014 Available at: http://www.ipcc.ch/pdf/assessment-report/ar5/wg3/ipcc_wg3_ar5_chapter11.pdf

21. West P C, Gibbs H K, Monfreda C, Wagner J, Barford C C, Carpenter S R et al. Trading carbon for food: global comparison of carbon stocks vs. crop yields on agricultural land Proc. Natl. Acad. Sci. U. S. A. 2010; 107: 19645–8. doi: 10.1073/pnas.1011078107

22. Veldman JW, Overbeck GE, Negreiros D, Mahy G, Le Stradic S, Fernandes GW, Durigan G, Buisson E, Putz FE, Bond WJ. Where tree planting and forest expansion are bad for biodiversity and ecosystem services. BioScience. 2015 Oct 1;65(10):1011-8. doi: 10.1093/biosci/biv118

23. Clark T S and Linzer D A. Should I Use Fixed or Random Effects? Polit. Sci. Res. Methods. 2015;3: 399–408. doi: 10.1017/psrm.2014.32

24. Hertel T W, Ramankutty N and Baldos U L C. Global market integration increases likelihood that a future African Green Revolution could increase crop land use and CO2 emissions. Proc. Natl. Acad. Sci. U. S. A. 2014;111: 13799–804. doi:

10.1073/pnas.1403543111

25. Blaustein-Rejto D, Blomqvist L, McNamara J and De Kirby K. Achieving Peak Pasture: Shrinking Pasture's Footprint by Spreading the Livestock Revolution. The Breakthrough Institute. 2019.

Supporting information

Formatted: Heading 1

S1 ~~Text~~File: Supplementary methods, figures and tables.
