# Supplementary material for: Carbon opportunity cost increases carbon footprint advantage of grain-finished beef
Source: PLoS One. 2023 Dec 13;18(12):e0295035. doi: 10.1371/journal.pone.0295035 (PMC10718409; doi:10.1371/journal.pone.0295035)
Supplement: Attachment — Submitted filename: reviewer_response_clean.docx [file pone.0295035.s002.docx]

# Journal requirements:

When submitting your revision, we need you to address these additional requirements.

1.  Please ensure that your manuscript meets PLOS ONE's style requirements, including those for file naming. The PLOS ONE style templates can be found at

<https://journals.plos.org/plosone/s/file?id=wjVg/PLOSOne_formatting_sample_main_body.pdf>  and

<https://journals.plos.org/plosone/s/file?id=ba62/PLOSOne_formatting_sample_title_authors_affiliations.pdf>

We have ensured that the manuscript, including file naming, meets the requirements. We added a Supplementary Information section heading to the manuscript, changed the supplementary figure and table references from the style “Table S1” to “S1 Table”, removed the “short title”, and we moved Table 2 and the caption for Figure 2 to appear directly after the paragraph in which they are first cited.

2. We note that Figure S1 in your submission contain [map/satellite] images which may be copyrighted. All PLOS content is published under the Creative Commons Attribution License (CC BY 4.0), which means that the manuscript, images, and Supporting Information files will be freely available online, and any third party is permitted to access, download, copy, distribute, and use these materials in any way, even commercially, with proper attribution. For these reasons, we cannot publish previously copyrighted maps or satellite images created using proprietary data, such as Google software (Google Maps, Street View, and Earth). For more information, see our copyright guidelines: <http://journals.plos.org/plosone/s/licenses-and-copyright>.

We require you to either (1) present written permission from the copyright holder to publish these figures specifically under the CC BY 4.0 license, or (2) remove the figures from your submission:

A. You may seek permission from the original copyright holder of Figure S1 to publish the content specifically under the CC BY 4.0 license.

We recommend that you contact the original copyright holder with the Content Permission Form (<http://journals.plos.org/plosone/s/file?id=7c09/content-permission-form.pdf>) and the following text:

“I request permission for the open-access journal PLOS ONE to publish XXX under the Creative Commons Attribution License (CCAL) CC BY 4.0 (<http://creativecommons.org/licenses/by/4.0/>). Please be aware that this license allows unrestricted use and distribution, even commercially, by third parties. Please reply and provide explicit written permission to publish XXX under a CC BY license and complete the attached form.”

Please upload the completed Content Permission Form or other proof of granted permissions as an "Other" file with your submission.

In the figure caption of the copyrighted figure, please include the following text: “Reprinted from [ref] under a CC BY license, with permission from [name of publisher], original copyright [original copyright year].”

B. If you are unable to obtain permission from the original copyright holder to publish these figures under the CC BY 4.0 license or if the copyright holder’s requirements are incompatible with the CC BY 4.0 license, please either i) remove the figure or ii) supply a replacement figure that complies with the CC BY 4.0 license. Please check copyright information on all replacement figures and update the figure caption with source information. If applicable, please specify in the figure caption text when a figure is similar but not identical to the original image and is therefore for illustrative purposes only.

The following resources for replacing copyrighted map figures may be helpful:

USGS National Map Viewer (public domain): <http://viewer.nationalmap.gov/viewer/>

The Gateway to Astronaut Photography of Earth (public domain): <http://eol.jsc.nasa.gov/sseop/clickmap/>

Maps at the CIA (public domain): <https://www.cia.gov/library/publications/the-world-factbook/index.html> and <https://www.cia.gov/library/publications/cia-maps-publications/index.html>

NASA Earth Observatory (public domain): <http://earthobservatory.nasa.gov/>

Landsat: <http://landsat.visibleearth.nasa.gov/>

USGS EROS (Earth Resources Observatory and Science (EROS) Center) (public domain): [http://eros.usgs.gov/#](http://eros.usgs.gov/)

Natural Earth (public domain): <http://www.naturalearthdata.com/>

We added a note in the caption for S1 Fig that the map data is from Natural Earth, a public domain source.

Additional Editor Comments :

In general, the manuscript presents an adequate structure with some typographical errors. **It is necessary that you can add additional information and explain more extensively some techniques of statistical analysis which has been requested by the reviewers, this will strengthen the manuscript**.

As detailed below in the responses to the reviewers, we have addressed the typographical errors, added additional information, and explained more extensively the techniques of statistical analysis.

# Review Comments to the Author

## **Reviewer** #1

Check the title.

We have checked the title. If the reviewer suggests or requires any changes to the title prior to publication, we will gladly consider them.

40-43. Cite this part.
We added a citation that was also used elsewhere in the manuscript.

47-49. Cite this part.

We edited to make the claim broader and added a supporting citation that was also used elsewhere in the manuscript.

89-94. These results should not be there.

We removed this paragraph and the results presented in the following paragraph (originally lines 98-100).

130, 154, 157. Is it possible to reference the equations?

Equations 1,2 and 3 were originally referred to in the text. We added another reference to equation 3 directly above it in line 156.

281 & 312. Please place figure title below the Fig.

We did not alter the figure titles or their placement. PLOS One’s [submission guidelines](https://journals.plos.org/plosone/s/submission-guidelines#loc-figures-and-tables) requested that we place the figure title in the text, but only include the figure as a separate file. “Figure captions must be inserted in the text of the manuscript, immediately following the paragraph in which the figure is first cited (read order).”

320. Please include all the definitions below the table. The table must be self explanatory.

We added definitions of the acronyms used, and also a note explaining that numbers in parentheses are standard errors.

Figure S1. There are no white circles, and the map has no color

We apologize for this error and thank you for calling attention to it. There appears to have been an issue with the image file type that resulted in it displaying incorrectly. We have replaced the figure with another image type that should prevent this problem.

Recommendation: you may consider placing different types of dots on figure 2. This can help colorblind (daltonic) people with their understanding.

Thank you. We changed the color palette to a more colorblind-friendly palette and also changed the dot/shape type to better differentiate.

## Reviewer #2

The paper conducts a pairwise comparison of pasture-finished operations and grain-finished operations. Also, the authors show that land-use intensity and carbon footprint are positively correlated. Thus, the paper concerns a fundamental and interesting research problem.

However, I have some recommendations for the authors:
1. Materials and methods.
a. Pairwise comparison: How did you collect the data? Table S8 shows information about 20 studies included in the pairwise comparison; how were the studies selected? It is necessary to add this information.

We added clarification in lines 194-196, noting that in addition to collecting data from studies listed in Poore and Nemecek (2018) and Stanley et al. (2020), “Systems were selected for inclusion if they were in the same subnational region or country, if the study was national in scope, and reported in the same study or within two studies by the same primary author.

b. Regression analysis: How many observations did you use? Are the units 72 operations or 24 studies? Is it a cross-section regression, or is it panel data? (In this case, which are the individuals, and which is the time?) Where are the results of the Hausman test? Is it possible to use the Hausman test in a cross-section model? It is important to explain which model was estimated and what type of data was used.

We added clarification in lines 204-206 that it is a “cross-section regression analysis of beef production operations. We include 72 operations from life-cycle assessments that report geographic coordinates, including a total of 24 studies in 12 countries.”

We clarified in the regression equation and the explanation of it (in lines 220-227) that it includes a random intercept at the country-level.

We have also revised the description (lines 228-232) of why we chose this specification. Clark and Linzer (2015) and Bell et al. (2019) suggest against using the Hausman test as it has low statistical power, and so we no longer justify our choice based on it. Gelman & Hill 2007 (p246) provide advice “to always use multilevel modeling (“random effects”)”. This is what we do in the paper, presenting similar results with a fixed effect specification in the SI. Clark and Linzer (2015) further supports this choice, writing that when “units are relatively similar to one another on average—the choice of random versus fixed effects only matters at extremely high levels of correlation between the independent variable and the unit effects.” The units (countries) in our dataset are relatively similar; the independent variable, log(LUI), has a mean standard deviation within countries of 0.629 and a mean standard deviation between countries of 0.892. Further, we have little reason to believe there are extremely high levels of correlation between the independent variable and unit effects; there is a correlation between country effects and log(LUI) of -0.36 with a random effects model and of. -0.35 with a fixed effect model.

Clark, T. S., & Linzer, D. A. (2015). Should I use fixed or random effects?. Political science research and methods, 3(2), 399-408.

Bell, A., Fairbrother, M., & Jones, K. (2019). Fixed and random effects models: making an informed choice. Quality & quantity, 53(2), 1051-1074

Gelman, A., & Hill, J. (2007). Data analysis using regression and multilevel/hierarchical models. Cambridge university press.

2. Results and discussion
a. Pairwise comparison: This section describes the main findings from the t-test, but it seems that the numbers in table S3 are different from the text. Why didn’t you compare the findings from the nonparametric test with the t-test?

Lines 294-299 in this section describe the percentage difference in values for the grain-and pasture-finished groups calculated from the t-test. Table S3 showed the other results from the t-test (the absolute difference and p-values). Thank you for calling attention to the fact that the table did not include the values in the main text. We have added the percentage difference values to the table.

We have also added description of the results from the nonparametric test to the main text and compared the results to those from the t-test (lines 299-302).

If your data was collected from studies, why didn’t you use meta-analysis inside the t-test? I suggest explaining how the studies were selected and considering making a meta-analysis.

We added description to the methods section, lines 194-196, of how the studies in the pairwise comparison were selected.

While we believe that conducting a meta-analysis of pasture- and grain-finished life cycle assessments published to-date would be valuable, we do not believe it would be accurate to describe any component of our study as a meta-analysis, even with adjustments to the statistical methods. All but one of the studies we use in our analysis are from a recent meta-analysis, Poore & Nemecek (2018), that standardized land-use intensity and production emission values across studies through a comprehensive gap-filling and standardization process described in that paper and its supplementary information. We took this approach instead of collecting an original set of life-cycle assessment studies based on novel eligibility criteria and an original, systematic review of literature, as would typically be done in a meta-analysis.

In addition, each beef operation in our dataset has only a single number associated with it (for each variable like production emissions) rather than a statistic estimated, from a sample, with a standard error. These statistics, or data from which they could be derived, would be required for typical meta-analysis methods e.g. to conduct a meta-analysis of mean differences.

b. Regression analysis. Were the assumptions of the model validated?

We appreciate the reviewers’ questions about the validity of the regression analysis results. We assessed whether the results are robust to violations of our primary assumptions.

It is commonly suggested that a key assumption for a regressions with a random-effects specification is that the independent variable and covariates not be corelated with the unit effects. However, as Clark and Linzer (2015) note, “it does not follow that *any* correlation between the covariates and the unit effects implies that fixed effects should be preferred. What should be judged instead is how much bias is created, and how much variance would be introduced by using fixed effects instead.” Based on the guidelines in this paper and our interest in estimating the average relationship between land use intensity, emissions, and total carbon footprint—rather than specific unit(i.e. country) effects—we report results from the random-effects specification in the main text. As noted above, there is not an extreme level of correlation between the independent variable and unit effects.

Yet if one nevertheless believes the assumptions for a random-effects specification *are* violated, we report results with a fixed-effect specification in Table S5 in the supplementary information. As we write in line 332 in the main text, “Regressions with… country fixed-effects specifications generate similar results.” In fact, the results from the two models are nearly indistinguishable. The fixed-effect specification generates an estimate of 0.48 when the dependent variable is PEM (0.48 with random effects), 0.34 when the dependent variable is PEM+SCS (0.32 with random effects), and 0.92 when the dependent variable is PEM+SCS+COC (0.90 with random effects). These estimates all have a p-value less than 0.01.

Are the same results if the authors add other variables (control)?

As line 333 in the main text notes, the “Results are robust to other specifications and assumptions checked” as detailed in Table S6 in the SI. We test whether the results are robust to different methods of calculating carbon opportunity cost and different assumptions for carbon sequestration rates per hectare. This robustness check confirms a positive and statistically significant (p<0.01) relationship between land-use intensity and total carbon footprint (PEM+SCS+COC).

We do not consider it necessary to add variables to the regression because we are not trying to identify the causal “effect” of land-use intensity. Rather we limit our analysis to estimating and reporting the statistical relationship between land-use intensity and different measures of the carbon footprint of beef production operations. In addition, most of the variables we could potentially add as controls are measures of resource use or environmental impact (e.g. freshwater withdrawal, eutrophication potential, acidification potential) that are not independent from our independent variable, land-use intensity. If the reviewer still has concerns about the validity of the results or would like to see an analysis with specific variables included, we are happy to provide additional information or analysis.

Are the number of observations enough to estimate this kind of model?

Given that estimates from the regression analyses with our preferred specifications (and from all the robustness checks) have p-values less than 0.01, we have no concerns about the number of observations being enough to estimate this kind of model. Our results demonstrate that that number of observations and the variability of values is sufficient to estimate this model in a way that rejects the null hypothesis of the slope coefficient being equal to zero.

# Reviewer #3

This paper presents a rather complete comparison of the carbon footprint of grain-finished and pasture-finished beef production Systems, taking into account production emissions, soil carbon sequestration and carbon opportunity cost. The study use global information from 16 countries and several model to estimate emissions are considered. It is generally well written, with very few typing errors. The statistical methods used are correct and the authors took into account assumptions that make them valid. I recommend publishing it once a few minor corrections have been done.

Minor corrections:
Line 36. Replace “ kg” by “ kilograms”
Lines 76-76 and 80. Replace “et al” by “et al.”
Line 107 . Replace “ kg” by “ kilograms”
Line 109 111 113 Replace “et al” by “et al.”
Line 117 Replace “&” by “and”
Line 121 Replace “ kg” by “ kilograms” and eliminate “(ha)”. Universal acronyms like “ha” should not be defined
Line 125 Replace “et al” by “et al.”
Line 135 Replace “0.28 Mg carbon (C) ha-1 yr-1 “ by “0.28 Mg C ha-1 yr-1 “
Line 156 Replace “Where” by “where”
Line 162 Replace “one kg” by “one kilogram” or by “1 kg”
Lines 164 and 167 Replace “et al” by “et al.”
Line 172 Replace “ kg” by “ kilograms”
In lines 70, 76, 80 and 81 among others you write the author’s name, year, and the number of citation between brackets, but in several lines like 176, 189, 239, 259 (and more) the number in brackets is absent.
Line 176 Replace “et al” by “et al.”
Line 206 “where” without capital letter
Line 237 Replace “MgC” by “Mg C”
Line 239 Replace “et al” by “et al.”
Line 254 and 261. Replace “kg CO2e/kg” by “kg CO2e kg-1”
Line 259 Replace “et al” by “et al.”
Line 264 Table 1. Eliminate the dot at the end of table title
Line 265 Replace “ kg” by “ kilograms”
Line 320 Table 2. explain what the numbers in parentheses are
Line 353 Replace “et al” by “et al.”
Line 362 Replace “et al” by “et al.”

Line 482-483 Replace Agricultural Systems by the abbreviation Agric. Syst.
Table S4: Replace “kgCO2e kg-1” by “kg CO2e kg-1”

We made the above minor corrections.
