# Supplementary material for: Carbon opportunity cost increases carbon footprint advantage of grain-finished beef
Source: PLoS One. 2023 Dec 13;18(12):e0295035. doi: 10.1371/journal.pone.0295035 (PMC10718409; doi:10.1371/journal.pone.0295035)
Supplement: Attachment — Submitted filename: Comments.docx [file pone.0295035.s004.docx]

It is submitted that no reportable issue is found in the manuscript with reference to results and discussion, but there are some minor corrections required specifically in the references format.

Ensure that all citations in the text are accurately matched with the corresponding entries in the References section

Standardize the citation style throughout the manuscript according to the journal formate

Verify that all the references listed are cited within the main text, and vice versa

Make sure the reference list is complete and does not contain any duplicates
